# Is there an optimum level of diversity in utilization of genetic resources?

**DOI:** 10.1007/s00122-017-2959-4

**Published:** 2017-08-05

**Authors:** Manfred Mayer, Sandra Unterseer, Eva Bauer, Natalia de Leon, Bernardo Ordas, Chris-Carolin Schön

**Affiliations:** 1 Plant Breeding, TUM School of Life Sciences Weihenstephan, Technical University of Munich, 85354 Freising, Germany; 2 Department of Agronomy, University of Wisconsin-Madison, Madison, WI USA 53706; 3 Misión Biológica de Galicia, Spanish National Research Council (CSIC), 36080 Pontevedra, Spain

## Abstract

*****Key message***:**

**Capitalizing upon the genomic characteristics of long-term random mating populations, sampling from pre-selected landraces is a promising approach for broadening the genetic base of elite germplasm for quantitative traits.**

**Abstract:**

Genome-enabled strategies for harnessing untapped allelic variation of landraces are currently evolving. The success of such approaches depends on the choice of source material. Thus, the analysis of different strategies for sampling allelic variation from landraces and their impact on population diversity and linkage disequilibrium (LD) is required to ensure the efficient utilization of diversity. We investigated the impact of different sampling strategies on diversity parameters and LD based on high-density genotypic data of 35 European maize landraces each represented by more than 20 individuals. On average, five landraces already captured ~95% of the molecular diversity of the entire dataset. Within landraces, absence of pronounced population structure, consistency of linkage phases and moderate to low LD levels were found. When combining data of up to 10 landraces, LD decay distances decreased to a few kilobases. Genotyping 24 individuals per landrace with 5k SNPs was sufficient for obtaining representative estimates of diversity and LD levels to allow an informed pre-selection of landraces. Integrating results from European with Central and South American landraces revealed that European landraces represent a unique and diverse spectrum of allelic variation. Sampling strategies for harnessing allelic variation from landraces depend on the study objectives. If the focus lies on the improvement of elite germplasm for quantitative traits, we recommend sampling from pre-selected landraces, as it yields a wide range of diversity, allows optimal marker imputation, control for population structure and avoids the confounding effects of strong adaptive alleles.

**Electronic supplementary material:**

The online version of this article (doi:10.1007/s00122-017-2959-4) contains supplementary material, which is available to authorized users.

## Introduction

Maize (*Zea mays* L. ssp*. mays*) landraces are a rich source of untapped allelic variation, but efficient strategies for exploring their genetic diversity are lacking. The successful use of landraces for improving elite germplasm has been hampered by insufficient genetic and phenotypic information and their heterogeneous and heterozygous nature (Sood et al. 2014). Linking genotypes to meaningful phenotypes by genome-enabled studies will pave the way for accessing the native diversity of landraces in a targeted way (McCouch et al. 2013; Tanksley and McCouch 1997). The success of these studies strongly depends on the choice of genetic material.

Genome-enabled studies with landrace material have successfully investigated crop evolution (Hufford et al. 2012; Matsuoka et al. 2002; van Heerwaarden et al. 2011), genomic signals and marker-trait associations for adaptation to different environments (Romero Navarro et al. 2017; Takuno et al. 2015) as well as the effects of rare alleles (Krakowsky et al. 2008). As such studies capitalize on maximizing diversity, mostly few individuals are sampled from many landraces covering a wide range of geographic regions. For the improvement of elite germplasm, an alternative approach might be more suitable, namely sampling many individuals from few pre-selected landraces. This sampling strategy comes at the expense of diversity, but might be advantageous for identifying novel alleles adapted to a specific set of environments and to the genetic background of a target elite breeding pool (Goodman 1999; Tarter and Holland 2006). Pre-selecting a representative set of landraces facilitates collection of meaningful phenotypes in the given environments and increases the incorporation efficacy of favorable alleles by reducing the risk of unexpected allelic effects (Lonnquist 1974; Sood et al. 2014). For allogamous crops such as maize, it has been shown that a large proportion of the molecular and phenotypic variation can be found within individual populations, whereas differences between major groups of landraces account only for a small proportion of the total variation (Sood et al. 2014; Vigouroux et al. 2008). In addition, the within-landrace sampling approach capitalizes upon the genomic characteristics of long-term random mating populations such as absence of hidden population structure and consistency of linkage phases. These factors can increase the accuracy and efficacy of genome-enabled approaches, such as genome-wide association studies (GWAS) and genomic selection. Thus, we hypothesize that in studies aiming at gene discovery or genomic selection based on landrace-derived material, an optimum rather than a maximum level of diversity might be beneficial. The comprehensive sampling of diversity within a few pre-selected landraces can be especially promising if the focus lies on the improvement of elite germplasm for quantitative traits. Recently, different strategies have been proposed for accessing the native diversity of landraces (Gorjanc et al. 2016; Melchinger et al. 2017) but a comprehensive comparison of within- and across-landrace estimates of genomic parameters with impact on the power of genome-enabled approaches has been lacking so far.

In this study, we analyzed genetic diversity, population structure, linkage disequilibrium (LD) and persistence of linkage phase within and across 35 European maize landraces with more than 20 individuals per landrace genotyped at high density. We investigated the effect of varying the number of sampled landraces and individuals per landrace on these parameters and give practical recommendations for assembling datasets for genome-enabled studies. We extended our analyses to Central and South American landraces of the Seeds of Discovery (SeeD) project (http://seedsofdiscovery.org) to assess the genetic diversity of European landraces in a broader context.

## Materials and methods

### Plant material and genetic data

**Fig. 1:**
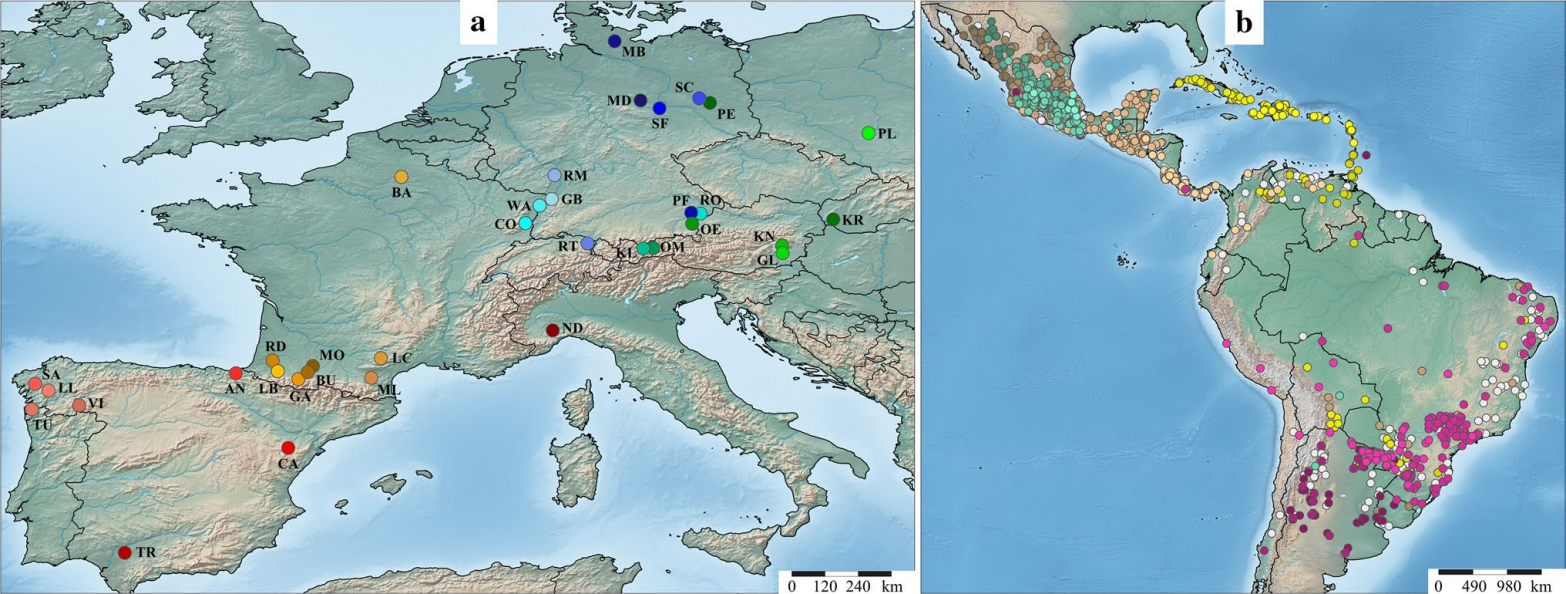
Geographical origin of European (**a**) and American (**b**) maize landraces investigated in this study. **a** North-eastern and south-western European landraces (Table S1) are colored in *blue*/*green* and *red*/*orange*, respectively. **b** The coloring of the American landraces from the SeeD project (Table S2) refers to different geographical macro regions: Caribbean islands (*yellow*), Central American and Mexican lowlands (*brown*), South America (*violet*-*red*) and Mexican highlands (*aquamarine*). The grouping of landraces was inferred by the analysis of population structure using ADMIXTURE with 16 genetic groups. Admixed landraces with less than 50% of their ancestry attributable to one of the 16 genetic groups are shown in *light gray*

We investigated 35 European maize landraces which were carefully chosen to cover a broad geographical region of Europe comprising different agro-ecological conditions (Fig. 1a). The panel included landraces with major historical relevance in terms of acreage (Oettler et al. 1976) and landraces from which important inbred lines of the European Flint elite breeding pool were derived (Messmer et al. 1992). Each landrace was represented by 22 to 48 plants, resulting in a total of 952 individuals. Name, abbreviation, geographical origin, seed source and the number of genotyped individuals (*n*_LR_) for each landrace are listed in Table S1. After DNA extraction following the protocol of Saghai-Maroof et al. (1984), each sample was genotyped with the 600k Affymetrix^®^ Axiom^®^ Maize Array (Unterseer et al. 2014). Markers designed to specifically differentiate between two Dent lines (Ganal et al. 2011) and indels were excluded. Analyses were performed based on markers assigned to the best quality class (Unterseer et al. 2014), with a call rate ≥0.9 and known physical position in the B73 reference sequence (AGP_v2; Chia et al. 2012). All individuals exhibited a call rate ≥0.9, consequently the dataset EU-Array consisted of 952 individuals and 516,797 SNPs.

The publicly available unimputed dataset of the SeeD maize GWAS panel (Hearne et al. 2014) of the International Maize and Wheat Improvement Center (CIMMYT) comprises 4710 individuals from 4020 Central and South American maize landrace accessions (with different CIMMYT germplasm IDs) and 955,120 markers generated by genotyping by sequencing (GBS; Elshire et al. 2011). The dataset was filtered for landraces with known geographical origin, bi-allelic SNPs with a minimum call rate of 0.8 and individuals with a minimum call rate of 0.8. Thus, dataset SeeD-GBS consisted of 3101 individuals from 2601 accessions (Fig. 1b) and 104,223 SNPs. The CIMMYT germplasm IDs and the number of individuals per accession are listed in Table S2. For comparing European and American landraces, the two datasets EU-OL and SeeD-OL were created, each comprising the 5045 SNPs which overlapped between EU-Array and SeeD-GBS. The distribution of SNPs in the different marker sets is shown exemplarily for chromosome 10 in Fig. S1. A summary of the different datasets is given in Table S3.

If not denoted otherwise, analyses within landraces were based on samples of 22 to 24 individuals (24 individuals were randomly sampled for *n*_LR_ > 24; Table S1) and for analyses across landraces individuals were randomly sampled under the side condition that each individual originated from a different landrace. Analyses were done using R version 3.0.1 (R Core Team 2013).

### Site frequency spectrum

The term site frequency spectrum (SFS) refers to the distribution of allele frequencies for a given set of SNPs. Let *f*_*i*_ be the proportion of SNPs with a derived allele frequency of *i*/*g* in a sample of *g* gametes. The (unfolded) SFS is then given by the vector *f* = (*f*_1_ *, f*_2_, …*, f*_*g−*1_). Following Nielsen and Slatkin (2013), the expected SFS under the standard neutral coalescence model with infinite sites mutations is given by:

1 $$E[f_{i} ] = \frac{1}{{i\mathop \sum \nolimits_{j = 1}^{g - 1 } \frac{1}{j}}}\quad i \, = \, 1, \, 2, \ldots ,g - 1.$$

Here, we calculated a folded SFS f* which describes the distribution of minor allele frequencies and is obtained by *f*_*i*_ * = *f*_*i*_ + *f*_*g−i*_ for *i* < *g*/2 and *f*_*i*_ * = *f*_*i*_ for *i* = *g*/2. For a given dataset, *g* gametes with non-missing genotype calls were randomly sampled per SNP, where *g* corresponds to 2*n* × *c* with *n* referring to the respective number of individuals and *c* to the minimum call rate (*c* = 0.8 and *c* = 0.9 for American and EU landrace datasets, respectively). For the estimation of the folded SFS, the number of minor alleles per SNP was averaged over 1000 random samples.

### Genetic diversity

Genetic diversity was assessed based on proportion of polymorphic markers (*PP*), nucleotide diversity (*π*) per marker (Nei and Li 1979) and haplotype heterozygosity (*H*; Nei and Tajima 1981). *H* was measured for sliding windows of 100 kb, with steps of 1 SNP and a minimum number of 5 SNPs per window. To obtain genome-wide estimates, mean *π* over all markers and mean *H* over all windows were calculated. Average deviation of genotype frequencies from Hardy–Weinberg expectations within populations was calculated using Weir and Cockerham’s *F*_is_ (Weir and Cockerham 1984). For dataset EU-Array, genetic diversity parameters and *F*_is_ were estimated within each landrace and for 1000 random samples of 24 individuals across landraces. To assess the effects of sample size on diversity estimates, the parameters were calculated for 24 randomly sampled as well as for all genotyped individuals within the five landraces with *n*_LR_ > 24. The results were compared between EU-Array and EU-OL to evaluate the effects of marker number and distribution. For datasets EU-OL, SeeD-OL and SeeD-GBS, diversity parameters and *F*_is_ were estimated based on 1000 random samples of 35 individuals across landraces. Using the R-package ade4 (Dray and Dufour 2007) version 1.6.2, an analysis of molecular variance (AMOVA; Excoffier et al. 1992) was performed to partition the total molecular variation of dataset EU-Array into within- and between-landrace components. Furthermore, AMOVA was used to estimate the proportion of the total molecular variance captured by groups of *l* landraces, with *l* = 1, 2, 3, 4, 5, 6, 7, 9, 18. For each *l*, landraces of dataset EU-Array, with 22 to 24 individuals per landrace (24 individuals were randomly sampled for *n*_LR_ > 24; Table S1), were randomly assigned to groups of *l* landraces, with the number of groups being the smallest integer ≥35/*l*. If 35 was not a multiple of *l* (for *l* = 2, 3, 4, 6, 9, 18), one group comprised only *l* − 1 landraces. For each *l*, we conducted 10,000 random repeats. Following Excoffier et al. (1992), significance for AMOVA and *F*_is_ was evaluated based on 1000 permutations, respectively.

### Population structure

To analyze the genetic relationship between individuals, an unrooted neighbor joining tree (NJT; Saitou and Nei 1987) was constructed and principal coordinate analysis (PCoA; Gower 1966) was performed, using the R-package ape (Paradis et al. 2004) version 3.4. NJT and PCoA were based on pairwise modified Rogers’ distances (MRD; Wright 1978) between individuals. NJT was constructed for dataset EU-Array. PCoA was calculated for each individual dataset as well as for a combined dataset based on SeeD-OL and one representative of each of the 35 landraces sampled from EU-OL. The correlations between MRD matrices obtained by datasets EU-Array/EU-OL and SeeD-GBS/SeeD-OL were evaluated using a Mantel test (Mantel 1967). PCoA patterns for the first three axes were compared between EU-Array and EU-OL and between SeeD-GBS and SeeD-OL via Procrustes analysis, using R-package ade4 (Dray and Dufour 2007) version 1.6.2. The software ADMIXTURE (Alexander et al. 2009) version 1.23 was used to analyze population structure. The algorithm implemented in ADMIXTURE assumes linkage equilibrium between SNPs, therefore, we pruned SNPs based on pairwise LD using the sliding window approach of PLINK (Purcell et al. 2007) version 1.7 with a window size of 50 SNPs, in steps of 5 SNPs and with an *r* ^2^ threshold of 0.8. For the estimation of the most likely number of genetic groups *K* in a given dataset a fivefold cross-validation (CV) approach was applied as implemented in ADMIXTURE. In dataset EU-Array we performed one run for each *K* varying from 1 to 25 and 20 runs with different seed settings for each *K* varying from 26 to 50. Additionally, for *K* = 35, 20 runs were conducted in a supervised mode, in which 35 genetic groups were pre-defined by choosing one individual per landrace as representative of the respective genetic group. In dataset SeeD-GBS, we performed 20 runs with different seed settings for each *K* varying from 1 to 25 and one run for each *K* varying from 26 to 50. For *K* = 35 (EU-Array) and *K* = 16 (SeeD-GBS) population structure according to the model with the lowest CV error of the respective 20 runs was visualized using a customized R-script.

### Linkage disequilibrium

Following Hill and Robertson (1968), LD was estimated as *r* ^2^. We calculated *r* ^2^ for pairs of SNPs with a maximum distance of 1 Mb and investigated the decay of *r* ^2^ with physical distance using non-linear regression according to Hill and Weir (1988). An *r* ^2^ of 0.2 was used as the threshold to obtain the physical LD decay distance. For EU-Array, mean *r* ^2^ and *r* ^2^ decay distance were estimated within each landrace and for 1000 random samples of 24 individuals across landraces. For datasets EU-OL, SeeD-OL and SeeD-GBS, mean *r* ^2^ and *r* ^2^ decay distance were estimated for 1000 random samples of 35 individuals across landraces.

For dataset EU-Array, interchromosomal LD was estimated for 24 individuals sampled from *l* = 1, 2, 3, 4, 6, 8, 12, 24 landraces, with an equal number of individuals per landrace and 10 random repeats per *l*. To obtain comparable results, SNPs were binned according to their minor allele frequency in the respective sample of individuals in steps of 0.05 and for each chromosome 100 SNPs were randomly sampled per bin. The resulting 1000 polymorphic SNPs per chromosome were used for the calculation of interchromosomal LD. The significance of higher fractions of marker pairs with *r* ^2^ > 0.2 across landraces (*l* > 1) compared to within landraces (*l* = 1) was assessed using the two-sided Wilcoxon rank sum test (Wilcoxon 1945) with Bonferroni correction.

The effect of sample size on LD estimates was evaluated by calculating LD decay distances within the five landraces of dataset EU-Array with *n*_LR_ ≥ 46 (Table S1). In addition to calculations including all individuals within the respective landrace, the number of individuals was varied from 5 to 45 in steps of 5. The effect of sample composition on LD estimates was assessed based on dataset EU-OL. LD calculations were performed for sampling schemes varying in the number of landraces *l* and the number of gametes *g* per landrace. In steps of 1, *l* varied from 1 to 35 and *g* from 1 to 44, as 44 was the minimum number of gametes per landrace in EU-OL. For each *g* × *l* combination, LD decay distances were averaged over 10 random samples. Calculations were performed for sampling schemes with *g* × *l* ≥ 12. To evaluate the effects of marker distribution on LD estimation, LD calculations for varying *g* and *l* were performed analogously for dataset EU-Array, with *g* and *l* varying in steps of 5.

To assess the persistence of linkage phase between landraces of dataset EU-Array, marker pairs were binned according to their physical distance in steps of 10 kb. For each bin and each pair of landraces, we calculated the correlation between the *r* values of the respective landraces and the proportion of marker pairs with equal phase (*PEP*), i.e. with equal sign of *r* (Technow et al. 2012). Both parameters were also estimated for 100 random samples of half of the individuals within each of the five landraces with *n*_LR_ ≥ 46 (Table S1) compared to the second half.

### Imputation and phasing

For AMOVA, population structure analyses using ADMIXTURE, and the estimation of *H*, *F*_is_, MRD and LD, missing genotype calls were imputed and the haplotype phase inferred using BEAGLE (Browning and Browning 2009) version 4.0 with default settings except for parameter *nsamples*, which was set to 50. Phasing and imputation for dataset EU-Array were done for each landrace separately, while for Seed-GBS they were performed based on the entire dataset. For datasets EU-OL and SeeD-OL haplotype information and imputed genotypes were extracted from EU-Array and SeeD-GBS, respectively.

#### **Availability of data and materials**

Genotype calls for the European and the SeeD datasets are available at figshare (https://doi.org/10.6084/m9.figshare.4789414.v1) and the CIMMYT Seeds of Discovery dataverse repository (http://hdl.handle.net/11529/10034; Hearne et al. 2014), respectively.

## Results

### Genetic diversity and population structure within and across European maize landraces

**Fig. 2:**
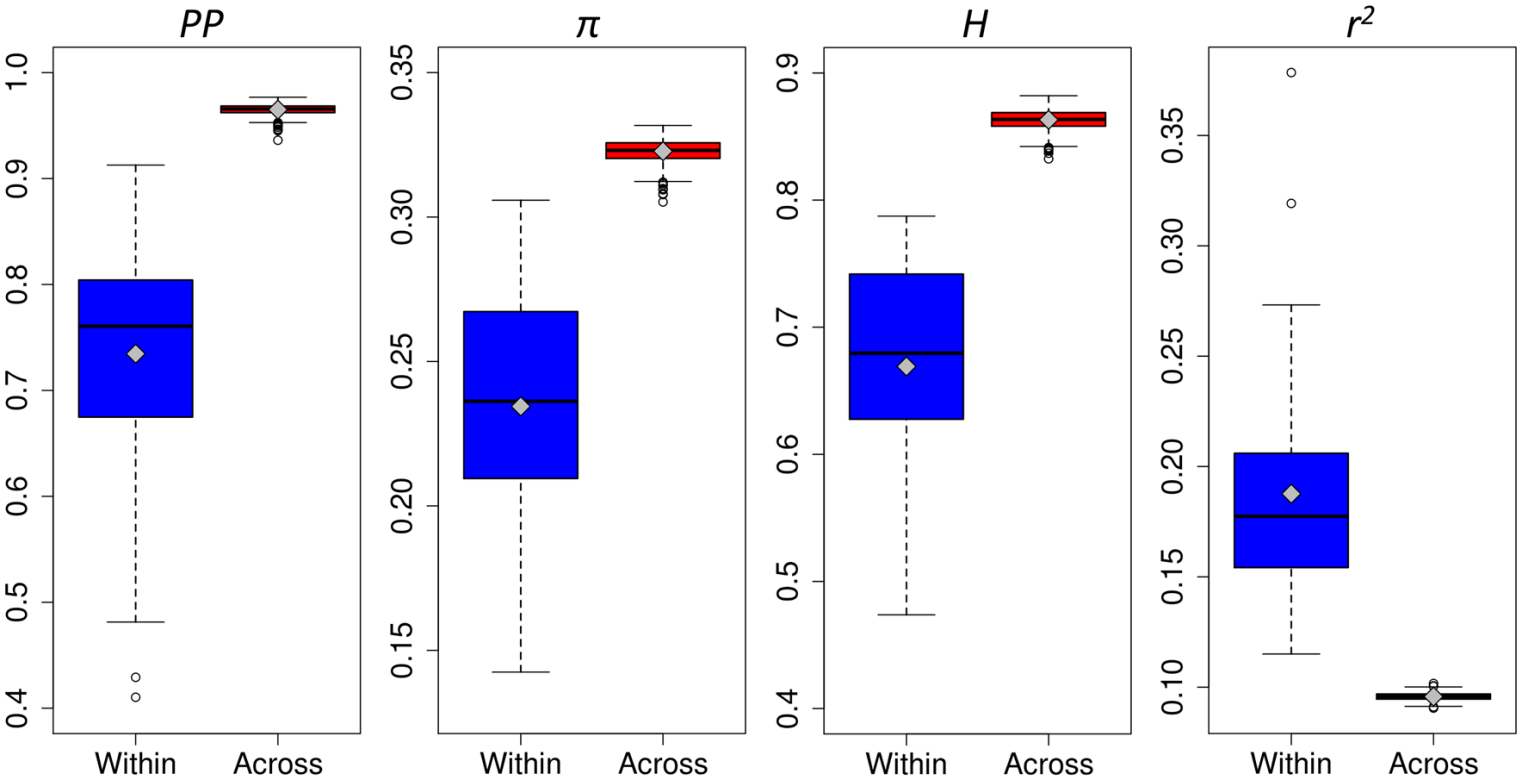
Genetic diversity and LD within and across European landraces. Proportion of polymorphic markers (*PP*), mean nucleotide diversity per marker (*π*), mean haplotype heterozygosity (*H*) and LD (mean *r* ^2^) were calculated based on dataset EU-Array. Boxplots represent values for samples of 22 to 24 individuals within each landrace (*blue*), and for 1000 random samples of 24 individuals across landraces (*red*). Boxplots show the upper and lower quartile, median (*horizontal bar*), mean (*gray diamond*) and whiskers (*vertical bars*) of the respective statistic. Points above and below the whiskers indicate values ±1.5 times the interquartile range

**Fig. 3:**
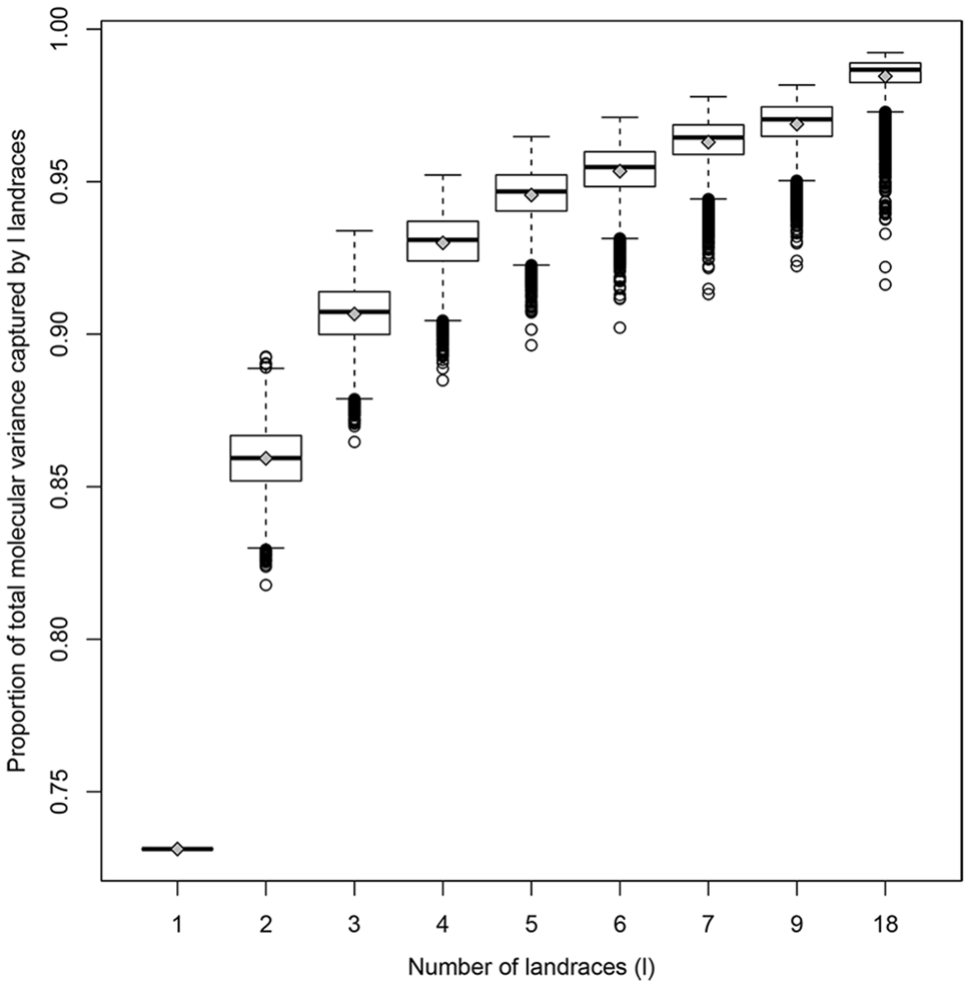
Proportion of the total molecular variance captured by different numbers of landraces. Landraces of EU-Array, with 22 to 24 individuals per landrace, were randomly assigned to groups comprising *l* landraces, with *l* = 1, 2, 3, 4, 5, 6, 7, 9, 18. The proportion of the total molecular variance of the panel of 35 landraces captured by groups of *l* landraces was estimated using AMOVA. Boxplots show the upper and lower quartile, median (*horizontal bar*), mean (*gray diamond*) and whiskers (*vertical bars*) for 10,000 random repeats per *l*. Points above and below the whiskers indicate values ±1.5 times the interquartile range

Dataset EU-Array comprised 952 individuals from 35 landraces (Fig. 1a; Table S1) and 516,797 SNPs with an overall call rate of 0.991. As expected for SNP array data, an excess of intermediate allele frequencies compared to the neutral expectation was observed (Fig. S2a), with an average minor allele frequency of 0.239. *PP*, *π* and *H* estimated across all 952 individuals were 0.999, 0.323 and 0.831, respectively. Landraces varied in their level of genetic diversity (Fig. 2; Table S4), with *PP*, *π*, and *H* ranging from 0.410 to 0.913, 0.142 to 0.306 and 0.474 to 0.787, respectively. Within landraces, the average levels of *PP*, *π*, and *H* were 0.735, 0.234 and 0.669, respectively. The average levels of *PP*, *π*, and *H* for 24 individuals randomly sampled across landraces were 0.965, 0.323 and 0.863, respectively. Genetic diversity parameters were on average higher within south-western compared to north-eastern European landraces (Table S5), though the three landraces with the highest *PP* and *π* values originated from Austria (GL, KN, OE; Table S4). For the five landraces with *n*_LR_ ≥ 46 (Table S1), estimates of diversity parameters for 24 randomly sampled individuals were comparable to levels observed including all individuals within these landraces (Table S6). Values of *F*_is_ were low and not significant for most landraces, ranging from −0.064 to 0.118 with a mean of 0.006 (Table S4). Five landraces showed a small but significant excess of homozygotes at the 0.05 significance level suggesting deviation from Hardy–Weinberg equilibrium due to inbreeding and/or population structure. AMOVA revealed that 73.1 and 26.9% of the total molecular variance of the 35 landraces originated from within and across landrace variation, respectively. The across landrace variance component was significant with *p* < 0.001. On average, ~95% of the molecular variation of the entire dataset (EU-Array) was already captured by groups of five landraces (Fig. 3).

The NJT revealed a clear genetic differentiation between the 35 landraces of dataset EU-Array, with a landrace-specific grouping of individuals (Fig. S3). Different levels of relatedness between landraces were indicated by the formation of geographical clusters, e.g. for landraces from the Alsace region (CO, GB, WA), from Galicia (LL, SA, TU, VI) and from the French Pyrenees (BU, GA, LB, MO, RD). Plotting the first and second principal coordinates (PCo) of the PCoA, a group of north-eastern European landraces was located in the first and fourth quadrant and a group of south-western European landraces in the second quadrant (Fig. S4). The third quadrant contained landraces from both regions. With the exception of ND, these landraces differed from the remaining landraces in their kernel morphology. While most landraces in dataset EU-Array showed typical Flint-like kernels with a thick, hard and vitreous outer layer, these landraces (CA, GL, KN, OE, PL, TR) displayed kernels with a small indentation, characteristic for Dent maize. Analogously, the NJT (Fig. S3) showed groups of Dent-like north-eastern European (GL, KN, OE, PL) and Dent-like Spanish landraces (CA, TR), respectively.

**Fig. 4:**
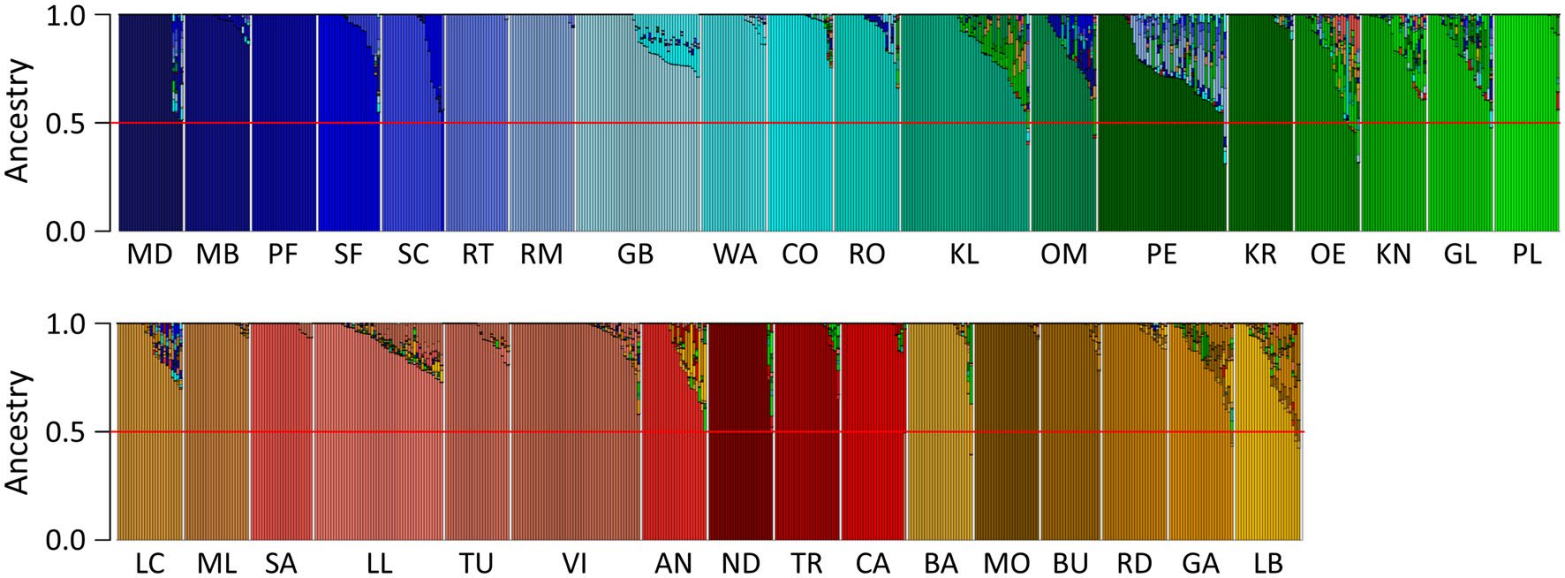
Population structure in European landraces. Population structure within dataset EU-Array was inferred using ADMIXTURE for 35 pre-defined genetic groups, colored according to Fig. 1a. Each *bar* represents one individual consisting of up to 35 *colors* according to their ancestry proportions attributable to each of the 35 genetic groups. The *red horizontal line* indicates an ancestry proportion of 50%. Landraces are ordered according to their position in the neighbor joining tree (Fig S3), with north-eastern and south-western European landraces at the *top* and *bottom*, respectively

Population structure of the landraces in the EU-Array panel was analyzed using the software ADMIXTURE. The most likely number of genetic groups *K* in the dataset could not be resolved unambiguously for *K* ranging from 1 to 50. CV errors decreased until *K* = 35, showed only minor differences for 35 ≤ *K* ≤ 40 and reached a plateau for *K* > 40 (Fig. S5). Therefore, given the 35 landraces in the panel, we chose one individual per landrace to represent the genetic composition of the respective landrace. A distinct separation of the 35 landraces was detected, whereas within landraces only limited evidence of population structure was observed (Fig. 4). The five Austrian landraces (GL, KL, KN, OE, OM) as well as AN, GA, LB and PE exhibited higher levels of admixture than the remaining landraces, but for almost all individuals more than 50% of their ancestry was attributed to the respective landrace.

### Linkage disequilibrium within and across European maize landraces

**Fig. 5:**
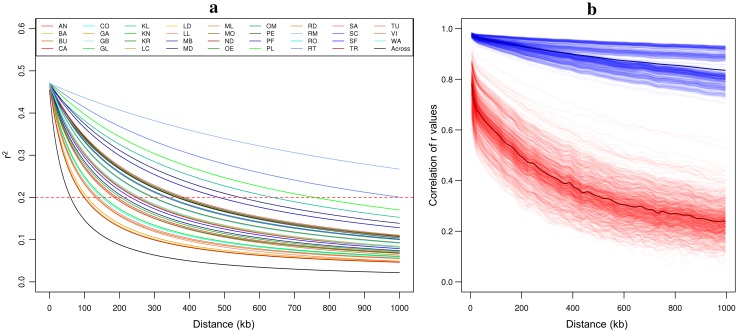
Decay of LD with physical distance and correlation of *r* within and across European landraces. **a** The decay of LD was estimated via non-linear regression using *r* ^2^ values for marker pairs within a maximum distance of 1 Mb. Based on dataset EU-Array, estimates for samples of 22 to 24 individuals within each landrace (*colored* according to Fig. 1a) and the mean over 1000 random samples of 24 individuals across landraces (*black*) are shown. The *red dashed line* indicates the threshold of *r* ^2^ = 0.2 for calculating the physical LD decay distance. **b** Cubic smoothing spline fits are shown for the correlation of *r* values between samples within (*blue*) and across (*red*) landraces as a function of physical distance, based on dataset EU-Array. For the within-landrace estimates, 100 times half of the individuals within each of the five landraces with *n*_LR_ ≥ 46 (Table S1) were randomly sampled and compared with the second half. Across-landrace estimates are based on pairwise comparisons of all 35 landraces. Mean values for within- and across-landrace estimates are shown in *dark blue* and *dark red,* respectively

Based on 22 to 24 individuals per landrace of dataset EU-Array, mean *r* ^2^ of SNP pairs within 1 Mb distance ranged from 0.115 to 0.379 with a mean of 0.188 (Table S4). Mean *r* ^2^ estimates calculated for 1000 random samples of 24 individuals across landraces were on average 0.096 and showed substantially less variation compared to within-landrace estimates, ranging from 0.091 to 0.102 (Fig. 2). Within landraces, LD decay distances ranged from 99 to 1809 kb with a mean of 342 kb (Fig. 5a; Table S4). For the majority of landraces, LD decay distance estimates were smaller than 500 kb, with north-eastern European landraces showing on average higher LD levels than south-western European landraces (Table S5). Compared to within-landrace estimates, smaller LD decay distances were obtained for samples across landraces (Fig. 5a; Table S4) ranging from 56 to 73 kb with a mean of 63 kb.

### Persistence of linkage phase within and across European maize landraces

The persistence of linkage phase for all pairwise comparisons of the 35 landraces of dataset EU-Array was evaluated based on the correlation of *r* values as well as *PEP*. For marker pairs with distances smaller than 10 kb, both parameters were high with a mean correlation of *r* values of 0.783 (Fig. 5b) and a mean *PEP* of 0.889 (Fig. S6). However, values of both parameters decreased rapidly with increasing physical distance between markers and reached moderate to low levels for marker pairs within distances of 990 to 1000 kb (mean correlation of *r* values = 0.238, mean *PEP* = 0.549). The persistence of linkage phase between pairs of landraces was associated with proximity of geographical origin and kernel type. The correlation of *r* values for marker pairs within 1 Mb distance was lowest for the comparison of the northern European Dent-like landrace PL and the southern European Flint-like landrace ND (0.298) and highest for a pair of two Flint-like German landraces (SC, SF; 0.747). *PEP* for marker pairs within 1 Mb distance was lowest for the comparison of the southern European Dent-like landrace TR and the northern European Flint-like landrace CO (0.564) and highest for a pair of two Dent-like Austrian landraces (GL, KN; 0.749). As expected, when comparing samples within each of the five landraces with *n*_LR_ ≥ 46 (Table S1), the two parameters were consistently high, for marker pairs within distances smaller than 10 kb (mean correlation of *r* values = 0.977, mean *PEP* = 0.972) as well as for marker pairs within distances of 990 to 1000 kb (mean correlation of *r* values = 0.836, mean *PEP* = 0.815; Fig. 5b; Fig. S6).

### Comparison of European and American landraces

To compare the molecular variation of the 35 temperate European landraces in this study with tropical Central and South American landraces and to assess specific properties of these datasets with respect to the use of different genotyping technologies, we extended our analyses to the SeeD maize GWAS panel. Dataset SeeD-GBS comprised 3101 individuals from 2601 accessions (Fig. 1b; Table S2) and 104,223 SNPs with an overall call rate of 0.907. Comparisons between European and American landraces were based on marker subsets of EU-Array and SeeD-GBS, each containing 5045 overlapping SNPs (datasets EU-OL and SeeD-OL). Compared to SeeD-GBS, an overrepresentation of intermediate allele frequencies pertained in these two subsets (Fig. S2b-d).

For each dataset, we estimated *PP*, *π*, *H*, mean *r* ^2^ and *r* ^2^ decay distance (Table S7) based on 1000 random samples of 35 individuals across landraces. All five parameters differed significantly between datasets (*p* < 0.001), as revealed by two-sided *t* tests with Bonferroni correction. The levels of *PP* and *π* were highest for EU-OL, slightly lower for SeeD-OL and lowest for SeeD-GBS. SeeD-GBS showed the highest level of *H* and only slightly lower values were observed for SeeD-OL, whereas *H* was lowest for EU-OL. Mean *r* ^2^ for marker pairs within 1 Mb distance and *r* ^2^ decay distances were highest for EU-OL, substantially lower for SeeD-OL and lowest for SeeD-GBS.

We used ADMIXTURE to identify major genetic groups within the American landrace panel (SeeD-GBS). CV errors decreased for the number of genetic groups *K* varying from 1 to 16 and reached a plateau for *K* > 16 (Fig. S7). Thus, we defined 16 genetic groups within SeeD-GBS. The resulting groups reflected the geographical origin of the respective landraces (Fig. S8). Five groups originated from the Mexican and Central American lowlands, four groups comprised landraces from the Mexican highlands, four groups referred to landraces from South America and three groups originated from the Caribbean islands and north-eastern South America. Individuals showed high levels of admixture, especially between geographically adjacent groups.

In the joint PCoA of SeeD-OL and one representative of each of the 35 European landraces sampled from EU-OL (Fig. S9), the first two PCos mainly separated South American from Mexican highland landraces with tropical Caribbean and Central American lowland landraces at the center. A group of north-eastern European Flint landraces was clearly separated from the American landraces. Part of the temperate European landraces, mainly from the south-west, grouped together with part of south-eastern South American landraces, but was clearly separated from the remaining groups. The genetic distance of European landraces to tropical Caribbean and Central American lowland landraces increased with increasing geographical distance to Mediterranean regions and was larger for Flint-like than for Dent-like landraces.

To evaluate the representation of population structure by the reduced marker sets EU-OL and SeeD-OL, we compared MRDs and PCoA between EU-OL and EU-Array and between SeeD-OL and SeeD-GBS, respectively. MRDs between individuals obtained by the respective reduced and full marker sets were highly correlated (correlation of 0.991 and 0.942 for EU and SeeD datasets, respectively; with a significance of *p* < 0.001; Fig. S10). Consistently larger MRDs were observed for SeeD-OL compared to SeeD-GBS. For the first three principle coordinates, the correlation-like statistic of Procrustes analyses was 0.994 for the comparison between EU-OL and EU-Array, and 0.991 between SeeD-OL and SeeD-GBS, respectively (*p* < 0.001).

### Influence of sample size, sample composition and marker distribution on LD estimates

**Fig. 6:**
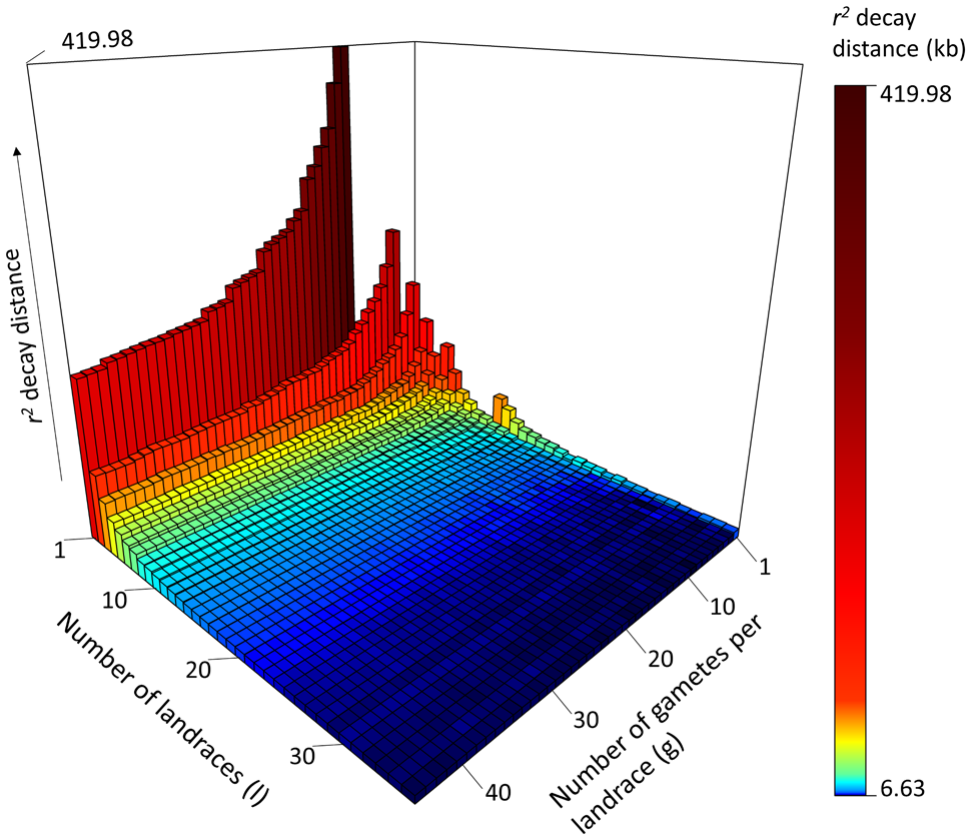
Effects of sample size and sample composition on the estimation of LD decay distances. Based on dataset EU-OL, LD decay distances were calculated using non-linear regression and an *r* ^2^ threshold of 0.2 for sampling schemes varying in the number of landraces *l* and the number of gametes *g* per landrace. *Bars* and *colors* represent the average LD decay distance for 10 random samples per *l* × *g* combination

Based on dataset EU-Array, we analyzed the effect of sample size on LD estimates by calculating LD decay distances for random samples of individuals within each of the five landraces GB, KL, LL, PE and VI with *n*_LR_ ≥ 46. For sample sizes smaller than 20 individuals (40 gametes), a strong increase in mean and variance of LD decay distance estimates was observed with decreasing sample size (Fig. S11). We also calculated LD decay distances for sampling schemes varying in the number of landraces *l* and the number of gametes *g* per landrace, based on dataset EU-OL. As expected, estimates of LD decay distance increased with decreasing total number of gametes (Fig. 6). For a given total number of gametes, LD decay distances were larger within landraces (*l* = 1) than across landraces (*l* > 1). For example, an LD decay distance of 174.3 kb was observed for 35 gametes sampled from one landrace in contrast to 8.3 kb when sampling 35 landraces with one gamete each. In general, LD decay distances decreased for increasing *l*, with the largest decrease observed for *l* from 1 to 10, and only marginal changes for *l* larger than 10. Analogously, LD calculations for varying *g* and *l* were performed for dataset EU-Array. A decrease in LD estimates with increasing *g* and *l* was also observed for EU-Array, but with substantially higher overall levels of LD compared to EU-OL (Fig. S12). The different levels of genome-wide LD estimates between EU-OL and EU-Array can be explained by differences in the distribution of markers, with EU-OL showing a higher marker density in telomeric regions compared to EU-Array (Fig. S1). However, LD estimates of landraces relative to each other were comparable between EU-OL and EU-Array (Table S6).

In admixed populations, LD can appear between unlinked markers due to differences in allele frequencies of subpopulations. To assess the extent of admixture-induced LD, we calculated interchromosomal LD for 24 individuals sampled from *l* = 1, 2, 3, 4, 6, 8, 12, 24 landraces. Overall, the proportion of interchromosomal marker pairs with *r* ^2^ > 0.2 was low (Table S8), but the Wilcoxon rank sum test revealed significantly higher proportions of marker pairs with *r* ^2^ > 0.2 across landraces (*l* > 1) than within landraces (*l* = 1).

## Discussion

When building GWAS discovery panels or training sets for genomic prediction from landraces, large data sets of several hundreds or even thousands of individuals are required to obtain sufficient power of QTL detection and high accuracy of prediction. Different sampling strategies can be devised depending on the aim of the study. When aiming at elucidating mechanisms of plant adaptation or discovering novel alleles for disease resistance or quality traits, maximizing the allelic diversity of the discovery panel is crucial. Thus, individuals might be sampled from many landraces covering a wide range of diversity with each landrace being represented by one or few individuals. An alternative strategy is to sample many individuals from each of a few pre-selected landraces, which might be especially promising for broadening the genetic diversity of elite material for quantitative traits.

In this study, we compared estimates of genomic parameters with impact on the power of genome-enabled approaches between different sampling strategies, using dense genotyping data from 35 European maize landraces with more than 20 individuals per landrace. We show for this unique set of landraces covering a wide range of eco-geographic conditions in the temperate maize growing regions of Europe that the majority of the landraces represented unstructured populations as indicated by low *F*_is_ values, a consistent landrace-specific grouping of individuals in NJT and PCoA, and high ancestry proportions of individuals attributable to their respective landrace (Fig. 4; Fig. S3–S4). With current advances in assembling complex genomes de novo (Unterseer et al. 2017) generating high-quality reference sequences that represent the diversity of a defined set of landraces is within reach. Given that linkage phases were highly consistent within landraces over fairly long genomic distances, imputation of missing genotypes from skim whole-genome sequencing should be possible with high accuracy for a broad range of allele frequencies. This should allow efficient characterization of haplotype variation within and across landraces.

Sampling individuals from a limited number of pre-selected landraces yields only slightly reduced levels of molecular diversity compared to sampling from the entire set of 35 European landraces. On average more than 70% of the total molecular variance present in the 35 landraces was found within landraces and about 95% was captured by samples of five landraces. Based on this high molecular variation, we can assume high genetic variation for quantitative traits of interest within a pre-selected set of landraces, which is in concordance with phenotypic investigations of landrace-derived material (Böhm et al. 2017; Wilde et al. 2010). LD levels within landraces were comparable to or lower than levels reported previously for diverse collections of temperate maize elite lines genotyped with the same array (Unterseer et al. 2014), thus yielding comparable mapping resolution in gene discovery studies. Moreover, mapping resolution for gene discovery can be increased by combining data from several landraces (Fig. 6). When sampling individuals from 10 landraces, LD decay distances of a few kb were observed, comparable to the level of the entire set of 35 European landraces and sufficiently low for candidate gene identification. Diversity and LD parameters varied between landraces with the majority of landraces retaining high levels of diversity and moderate to low levels of LD during their maintenance by farmers, their recollection and/or their preservation in gene banks. When adding a pre-screening step, the molecular and genetic variance in the data can be increased, as landraces deviating from expectations with respect to diversity, inbreeding or population structure can be excluded. Our results suggest that genotyping 24 individuals per landrace with 5k SNPs was sufficient for obtaining representative estimates of diversity and LD levels for each population (Fig. S11; Table S6). The usefulness of the data set can be further increased by evaluating a broad panel of landraces well adapted to a given target environment in the pre-screening step and by assuring that the selected landraces are segregating for target traits.

We found a gradually decreasing level of relatedness of European to Central and South American landraces with increasing geographical distance to Mediterranean regions (Fig. S9) consistent with previous observations (Dubreuil et al. 2006; Rebourg et al. 2003). This indicates that European landraces represent a broad spectrum of allelic variation, shaped by local adaptation to different agro-ecological zones. Haplotype diversity in the 35 European landraces was lower compared with the SeeD data but still sufficiently high to warrant high genetic variance for quantitative traits of interest. This was also confirmed by a recent study by Böhm et al. (2017) who described high levels of genetic variance for a suite of quantitative traits in doubled-haploid libraries derived from landraces of similar origin as those investigated in this study. While the haplotype based parameter *H* was presumably less affected by ascertainment bias than single SNP measures (Conrad et al. 2006), an enrichment of intermediate allele frequencies as well as an increase in *PP*, *π* and *r* ^2^ estimates indicated an overestimation of these parameters in the SeeD dataset when filtering for SNPs overlapping with the 600k array (Fig. S2; Table S7). Array-derived SNPs are restricted to the initial SNP discovery panel and affected by subsequent filtering steps, leading to an enrichment of intermediate allele frequencies compared to GBS-derived SNPs. As the array was optimized for temperate maize, *PP*, *π* and *r* ^2^ estimates were likely overestimated in European relative to American landraces. In both, the EU-Array and the SeeD-GBS datasets, SNPs were called using the B73 reference sequence and are, therefore, restricted to genomic regions present in B73. GBS-derived data depend on restriction enzyme cutting sites and hence are highly overrepresented in telomeric regions (Romay et al. 2013), as it was also observed in this study when comparing the distribution of SNPs between the Seed-GBS and EU-Array datasets. The differences in marker distributions were likely the main reason for the observed differences in genome-wide LD estimates between EU-Array and EU-OL (Fig. S1, S12) as the two datasets showed similar SFS (Fig. S2). Thus, comparisons of diversity parameters and LD between datasets analyzed with different genotyping technologies need to be interpreted with caution. However, inferences within the respective datasets of European or American landraces should be affected to a minor extent by these limitations and as can be seen from Fig. S9 the results of the PCoA obtained with the SNPs represented in the SeeD-OL dataset were consistent with those presented by Romero Navarro et al. (2017).

Within the European dataset, the grouping of the 35 landraces (Fig. S3–S4) with respect to their geographical origin and kernel type was clearly reflected in the genomic analyses. The level of interchromosomal LD induced by admixture was overall low, but, as expected, varied significantly depending on the sampling strategy (Table S8). However, when constructing data sets by sampling individuals from pre-selected landraces, the clear differentiation between populations allows a priori definition of subpopulations in statistical analyses to avoid false-positive marker-trait associations or inflation of prediction accuracies. In addition, when sampling a sufficiently high number of individuals within landraces, specific marker effects can be estimated using appropriate statistical models as suggested by Lehermeier et al. (2015).

Even though only one or few individuals were sampled from individual landraces in the SeeD-GBS data set, population structure was prevalent with 16 genetic groups mainly representing the geographic origin of the landraces (Fig. S8). With a high proportion of individuals exhibiting strong population admixture, accounting for population structure in the SeeD data set is challenging. Furthermore, the consistency of allelic effect estimates of samples of landraces covering a wide range of geographic regions with respect to a given target elite breeding pool warrants further research. It has been shown that strong correlations of geographic coordinates and specific adaptive traits persist in these data sets (Romero Navarro et al. 2017; Zhao et al. 2007). As these authors pointed out, disentangling associations of target traits from adaptation as well as estimation of genotype × environment interactions is difficult in highly diverse landrace collections. Thus, we conclude, that the incorporation of favorable alleles from landraces into elite germplasm can be expected to be most efficient if landraces are chosen not solely based on maximum allelic diversity but also with respect to a similar environmental adaptation and genomic background as the target elite breeding pool.

## Conclusions

We show that sampling a limited number of pre-selected landraces should provide high genetic variance for quantitative traits of interest and high mapping resolution in gene discovery. Absence of pronounced population structure within landraces and clear genetic differentiation between landraces allows a priori definition of subpopulations in statistical analyses and consistency of linkage phases facilitates genotype imputation and haplotype characterization. Thus, for broadening the genetic diversity of elite material for quantitative traits, we recommend capitalizing upon the genomic characteristics of long-term random mating populations and the genetic diversity within a pre-selected set of landraces adapted to a comparable environment as the target elite breeding pool.

### **Author contribution statement**

CCS, EB, SU, NdL and MM conceived the study and discussed the results; MM investigated genotypic data and performed analyses; CCS and BO acquired funding; BO contributed part of the Spanish landrace data; MM drafted the manuscript; CCS, EB and SU edited the manuscript; all authors read and approved the final manuscript.

## Electronic supplementary material

Below is the link to the electronic supplementary material.

Supplementary material 1 (PDF 414 kb)

Supplementary material 2 (PDF 9158 kb)

Supplementary material 3 (XLSX 43 kb)
