# Supplementary material for: Is there an optimum level of diversity in utilization of genetic resources?
Source: Theor Appl Genet. 2017 Aug 5;130(11):2283–95. doi: 10.1007/s00122-017-2959-4 (PMC5641276; doi:10.1007/s00122-017-2959-4)
Supplement: Supplementary file 1 — Supplementary material 1 (PDF 414 kb) [file 122_2017_2959_MOESM1_ESM.pdf]

Theoretical and Applied Genetics

Is there an optimum level of diversity in utilization of genetic resources?

Manfred Mayer[1], Sandra Unterseer[1], Eva Bauer[1], Natalia de Leon[2], Bernardo Ordas[3], Chris-Carolin Schön[1*]

[1] Plant Breeding, TUM School of Life Sciences Weihenstephan, Technical University of Munich, Freising, Germany

[2] Department of Agronomy, University of Wisconsin-Madison, Madison, Wisconsin, USA

[3] Misión Biológica de Galicia, Spanish National Research Council (CSIC), Pontevedra, Spain

[*]Corresponding author

Email address: chris.schoen@tum.de

Supplemental tables

Table S1 Overview of European landraces with landrace name, geographical origin, seed source and number of genotyped individuals (n_{LR}) per landrace.

Landrace name		Geographical origin				Seed source	n_{LR}
Full name	Abb.	Region[a]	Country	Latitude[b]	Longitude[b]		
Andoain	AN	SW	Spain	43.219	-2.020	CSIC[c]	24
Barisis	BA	SW	France	49.579	3.328	INRA[d]	24
Bugard	BU	SW	France	43.257	0.314	UH[e]	22
Castellote	CA	SW	Spain	40.799	-0.319	CSIC	24
Colmar	CO	NE	France	48.079	7.359	INRA	24
Gazost	GA	SW	France	43.032	0.007	INRA	24
Gelber Badischer Landmais	GB	NE	Germany	48.859	8.210	UH	46
Gleisdorfer	GL	NE	Austria	47.107	15.709	LfL[f]	24
Kemater Landmais Gelb	KL	NE	Austria	47.255	11.274	LfL	48
Knillis	KN	NE	Austria	47.253	15.694	LfL	24
Krajova c29	KR	NE	Slovakia	48.200	17.350	LfL	24
Lacaune	LC	SW	France	43.550	2.583	INRA	24
Lalin	LL	SW	Spain	42.661	-8.111	CSIC	48
Lucq de Bearn	LB	SW	France	43.287	-0.659	INRA	24
Mahndorfer	MD	NE	Germany	52.061	11.094	LfL	24
Maleksberger	MB	NE	Germany	53.982	10.253	LfL	24
Millette du Lauragais 2	ML	SW	France	43.072	2.381	INRA	24
Moncassin	MO	SW	France	43.454	0.479	INRA	24
Nostrano dell Isola	ND	SW	Italy	44.612	8.242	INRA	24
Oberhuber Martha	OM	NE	Austria	47.269	11.414	LfL	24
Oesterreichische Landsorte	OE	NE	Austria	48.058	12.769	LfL	24
Petkuser Ferdinand Rot	PE	NE	Germany	51.981	13.349	LfL	48
Pfarrkirchner	PF	NE	Germany	48.419	12.940	LfL	24
Polnischer Landmais	PL	NE	Poland	51.000	18.500	LfL	24
Rheintaler Monsheim	RM	NE	Germany	49.634	8.301	LfL	24
Rheintaler (St. Gallen)	RT	NE	Switzerland	47.424	9.371	UH	23
Rottaler	RO	NE	Germany	48.391	12.945	LfL	24
Roux de Chalosse	RD	SW	France	43.631	-0.828	INRA	24
Santiago	SA	SW	Spain	42.878	-8.545	CSIC	23
Schindelmeiser	SC	NE	Germany	52.131	13.000	UH	23
Strenzfelder	SF	NE	Germany	51.804	11.710	UH	23
Tremesino	TR	SW	Spain	37.396	-5.630	CSIC	24
Tuy	TU	SW	Spain	42.049	-8.647	CSIC	24
Viana	VI	SW	Spain	42.180	-7.113	CSIC	48
Wantzenau	WA	NE	France	48.650	7.817	INRA	24

[a] Two different macro regions were defined based on biogeographical regions of Europe, NE = north-eastern Europe, SW = south-western Europe.

[b] Geographical coordinates were assigned to each landrace according to their described collection site or growing region, based on information provided by institutions or personal communications (Bernado Ordas, CSIC; Barbara Eder, LfL). For PL, no information was available, thus coordinates were estimated.

[c] Consejo Superior de Investigaciones Científicas, Pontevedra/ Zaragoza, Spain.

[d] French National Institute of Agricultural Research, Paris, France.

[e] University of Hohenheim, Stuttgart, Germany.

[f] Bavarian State Research Institute for Agriculture, Freising, Germany.

Table S3 Summary of the datasets investigated in this study. The number of individuals (n_{Set}) and SNPs, the applied genotyping method, overall call rate as well as mean and median minor allele frequency (MAF) for each dataset are given.

Dataset	n_{Set}	SNPs	Genotyping	Call rate	Mean MAF	Median MAF
EU-Array	952	516,797	Array[a]	0.991	0.239	0.229
SeeD-GBS	3,101	104,223	GBS[b]	0.907	0.132	0.072
EU-OL	952	5,045	Array	0.994	0.241	0.232
SeeD-OL	3,101	5,045	GBS	0.925	0.234	0.229

[a] 600k Affymetrix® Axiom® Maize Array (Unterseer et al., 2014).

[b] Genotyping-by-sequencing (Elshire et al., 2011).

Table S4 Diversity statistics and LD of European landraces. Based on dataset EU-Array, the number of sampled individuals (n_{Sample}) included in a given calculation, proportion of polymorphic markers (*PP*), nucleotide diversity per marker (π), haplotype heterozygosity (*H*), the deviation of allele frequencies from Hardy-Weinberg equilibrium (F_{is}), mean pairwise r^2 for marker pairs with a maximum distance of 1 Mb and r^2 decay distance (kb) are given for each landrace and as average for samples across landraces.

**Sample**	**n_{Sample} [a]**	***PP***	**π**	***H***	**F_{is} [b]**		**Mean r^2**	**r^2 decay distance**
					value	sig		
AN	24	0.813	0.270	0.748	0.023	ns	0.156	193
BA	24	0.769	0.239	0.680	-0.008	ns	0.177	260
BU	22	0.674	0.225	0.679	-0.008	ns	0.190	298
CA	24	0.805	0.253	0.719	0.004	ns	0.174	247
CO	24	0.741	0.222	0.662	0.007	ns	0.138	150
GA	24	0.878	0.284	0.765	0.032	ns	0.118	108
GB	24	0.665	0.205	0.628	0.003	ns	0.202	259
GL	24	0.913	0.306	0.787	0.009	ns	0.141	156
KL	24	0.770	0.236	0.637	-0.064	ns	0.238	638
KN	24	0.903	0.301	0.764	0.047	ns	0.159	203
KR	24	0.774	0.249	0.680	0.003	ns	0.208	377
LB	24	0.880	0.288	0.777	0.034	ns	0.115	102
LC	24	0.760	0.221	0.627	0.005	ns	0.175	251
LL	24	0.883	0.288	0.773	-0.009	ns	0.159	132
MB	24	0.680	0.214	0.647	0.082	**	0.164	213
MD	24	0.599	0.152	0.506	-0.049	ns	0.246	545
ML	24	0.761	0.258	0.724	-0.010	ns	0.152	184
MO	24	0.725	0.246	0.687	0.029	ns	0.189	296
ND	24	0.676	0.198	0.611	0.010	ns	0.208	368
OE	24	0.908	0.297	0.766	0.034	ns	0.153	186
OM	24	0.786	0.227	0.635	0.088	**	0.197	333
PE	24	0.801	0.248	0.681	0.118	***	0.198	363
PF	24	0.481	0.154	0.523	0.014	ns	0.243	484
PL	24	0.657	0.208	0.621	0.076	*	0.273	757
RD	24	0.801	0.271	0.747	-0.005	ns	0.130	134
RM	24	0.410	0.147	0.474	0.091	**	0.379	1,809
RO	24	0.698	0.215	0.633	0.050	ns	0.189	294
RT	23	0.429	0.142	0.479	-0.018	ns	0.319	1,007
SA	23	0.743	0.264	0.709	0.010	ns	0.211	386
SC	23	0.606	0.194	0.592	-0.022	ns	0.204	340
SF	23	0.687	0.211	0.636	0.010	ns	0.170	233
TR	24	0.767	0.235	0.737	0.001	ns	0.115	99
TU	24	0.829	0.274	0.755	0.023	ns	0.134	139
VI	24	0.803	0.259	0.722	-0.006	ns	0.166	186
WA	24	0.634	0.204	0.609	0.027	ns	0.180	256
Mean within[c]	24	0.735	0.234	0.669	0.006	ns	0.188	342
Across 24[d]	24	0.965	0.323	0.863	0.288	***	0.096	63
Overall	952	0.999	0.323	0.831	0.276	***	0.075	50
GB	46	0.703	0.207	0.627	0.014	ns	0.159	205
KL	48	0.811	0.249	0.664	-0.050	ns	0.223	463
LL	48	0.910	0.290	0.776	0.003	ns	0.113	102
PE	48	0.831	0.242	0.666	0.109	***	0.188	306
VI	48	0.834	0.261	0.723	-0.010	ns	0.136	151

[a] For the five landraces (GB, KL, LL, PE, VI) with n_{LR} > 24, the parameters were calculated for 24 randomly subsampled individuals.

[b] For F_{is}, significance of permutation test is given by ns = non-significant, * = p-value < 0.05, ** = p-value < 0.01 and *** = p-value < 0.001.

[c] Mean over the 35 within-landrace estimates of the respective parameter.

[d] 24 individuals were randomly sampled across landraces. Mean values over 1,000 samples are shown.

Table S5 Comparison of diversity and LD between north-eastern and south-western European landraces. Proportion of polymorphic markers (*PP*), mean nucleotide diversity per marker (π), mean haplotype heterozygosity (*H*), mean r^2 for marker pairs with a maximum distance of 1 Mb and r^2 decay distances (kb) below 0.2 were calculated based on dataset EU-Array for samples of 22 to 24 individuals within each landrace. The mean and standard error are shown for 19 north-eastern and 16 south-western European landraces, respectively. The significance of the differences between the two groups, as revealed by two-sided Wilcoxon rank sum test, is indicated by ns = non-significant, * = p-value < 0.05, ** = p-value < 0.01 and *** = p-value < 0.001.

Region	*PP*	π	*H*	Mean r^2	r^2 decay distance
North-eastern	0.692	0.218	0.630	0.211	453
	(± 0.034)	(± 0.011)	(± 0.021)	(± 0.014)	(± 91)
South-western	0.785	0.255	0.716	0.160	211
	(± 0.016)	(± 0.006)	(± 0.012)	(± 0.008)	(± 23)
	*	*	**	**	**

Table S6 Effects of sample size and marker number/distribution on diversity statistics and LD. Based on datasets EU-Array and EU-OL, proportion of polymorphic markers (*PP*), nucleotide diversity per marker (π), haplotype heterozygosity (*H*), the deviation of allele frequencies from Hardy-Weinberg equilibrium (F_{is}), mean pairwise r^2 for marker pairs with a maximum distance of 1 Mb and r^2 decay distance (kb) are given for the five European landraces with more than 46 genotyped individuals. The parameters were calculated for 24 randomly subsampled individuals as well as for all individuals within the respective landrace. n_{Sample} indicates the number of sampled individuals included in each calculation.

**Dataset**	**Landrace**	n_{Sample}	***PP***	π	***H***	F_{is} [a]		**Mean** r^2	r^2 **decay distance**
						value	sig		
EU-Array 516,797 markers	GB	46	0.703	0.207	0.627	0.014	ns	0.159	205
	GB	24	0.665	0.205	0.628	0.003	ns	0.202	259
	KL	48	0.811	0.249	0.664	-0.050	ns	0.223	463
	KL	24	0.770	0.236	0.637	-0.064	ns	0.238	638
	LL	48	0.910	0.290	0.776	0.003	ns	0.113	102
	LL	24	0.883	0.288	0.773	-0.009	ns	0.159	132
	PE	48	0.831	0.242	0.666	0.109	***	0.188	306
	PE	24	0.801	0.248	0.681	0.118	***	0.198	363
	VI	48	0.834	0.261	0.723	-0.010	ns	0.136	151
	VI	24	0.803	0.259	0.722	-0.006	ns	0.166	186
EU-OL 5,045 markers	GB	46	0.695	0.202	0.485	0.009	ns	0.153	116
	GB	24	0.655	0.200	0.476	-0.003	ns	0.174	149
	KL	48	0.810	0.253	0.549	-0.054	ns	0.221	338
	KL	24	0.770	0.238	0.516	-0.072	ns	0.254	486
	LL	48	0.905	0.293	0.668	0.001	ns	0.105	46
	LL	24	0.877	0.292	0.658	-0.003	ns	0.119	59
	PE	48	0.830	0.241	0.548	0.096	***	0.183	208
	PE	24	0.805	0.246	0.561	0.102	**	0.196	238
	VI	48	0.840	0.263	0.617	-0.018	ns	0.129	79
	VI	24	0.805	0.262	0.619	-0.014	ns	0.141	90

[a] For F_{is}, significance of permutation test is given by ns = non-significant, * = p-value < 0.05, ** = p-value < 0.01 and *** = p-value < 0.001.

Table S7 Comparison of diversity and LD between European and American landraces. Proportion of polymorphic markers (*PP*), mean nucleotide diversity per marker (π), mean haplotype heterozygosity (*H*), mean r^2 for marker pairs with a maximum distance of 1 Mb and r^2 decay distances (kb) below 0.2 were calculated for 1,000 random samples of 35 individuals across landraces for datasets EU-OL, SeeD-OL and SeeD-GBS. The mean and standard error for these 1,000 samples are shown per dataset. The same parameters were calculated across all individuals within each dataset. n_{Sample} indicates the number of sampled individuals included in each calculation.

Dataset	n_{Sample}	*PP*	π	*H*	Mean r^2	r^2 decay distance
EU-OL	35	0.982	0.326	0.732	0.080	7.270
		(± 5.97E-5)	(± 3.54E-5)	(± 1.48E-4)	(± 2.53E-5)	(± 0.027)
SeeD-OL	35	0.949	0.314	0.778	0.043	0.143
		(± 1.71E-4)	(± 7.83E-5)	(± 2.07E-4)	(± 2.55E-5)	(± 3.36E-4)
SeeD-GBS	35	0.683	0.189	0.814	0.027	0.037
		(± 1.37E-4)	(± 8.98E-5)	(± 2.07E-4)	(± 1.29E-5)	(± 3.65E-5)
EU-OL	952	0.999	0.324	0.728	0.067	7.102
SeeD-OL	3,101	0.999	0.318	0.783	0.025	0.105
SeeD-GBS	3,101	0.975	0.191	0.819	0.006	0.009

Table S8 Interchromosomal LD within and across European landraces. SNPs were binned according to their minor allele frequency in the respective sample of individuals in steps of 0.05. For each chromosome and each bin, 100 SNPs were randomly sampled resulting in 1,000 SNPs per chromosome. Based on these SNPs, interchromosomal r^2 was calculated for 24 individuals sampled from l = 1, 2, 3, 4, 6, 8, 12, 24 landraces of dataset EU-Array with 10 random repeats per l. For each l, the mean and standard error of the percentage of marker pairs with $r^2 > 0.2$ are shown. The significance of larger fractions of marker pairs with $r^2 > 0.2$ across landraces ($l > 1$) compared to within landraces ($l = 1$) is indicated by ns = non-significant, * = p-value < 0.05, ** = p-value < 0.01 and *** = p-value < 0.001.

l	**Percentage $r^2 > 0.2$**
1	0.558 (± 0.050)
2	3.281 (± 0.853) ***
3	3.140 (± 0.541) ***
4	2.522 (± 0.451) ***
6	1.831 (± 0.274) ***
8	1.598 (± 0.116) ***
12	1.232 (± 0.068) ***
24	0.936 (± 0.048) ***
