# Supplementary material for: Is there an optimum level of diversity in utilization of genetic resources?
Source: Theor Appl Genet. 2017 Aug 5;130(11):2283–95. doi: 10.1007/s00122-017-2959-4 (PMC5641276; doi:10.1007/s00122-017-2959-4)
Supplement: Supplementary file 2 — Supplementary material 2 (PDF 9158 kb) [file 122_2017_2959_MOESM2_ESM.pdf]

Theoretical and Applied Genetics

Is there an optimum level of diversity in utilization of genetic resources?

Manfred Mayer[1], Sandra Unterseer[1], Eva Bauer[1], Natalia de Leon[2], Bernardo Ordas[3], Chris-Carolin Schön[1*]

[1] Plant Breeding, TUM School of Life Sciences Weihenstephan, Technical University of Munich, Freising, Germany

[2] Department of Agronomy, University of Wisconsin-Madison, Madison, Wisconsin, USA

[3] Misión Biológica de Galicia, Spanish National Research Council (CSIC), Pontevedra, Spain

[*]Corresponding author

Email address: chris.schoen@tum.de

Supplemental figures

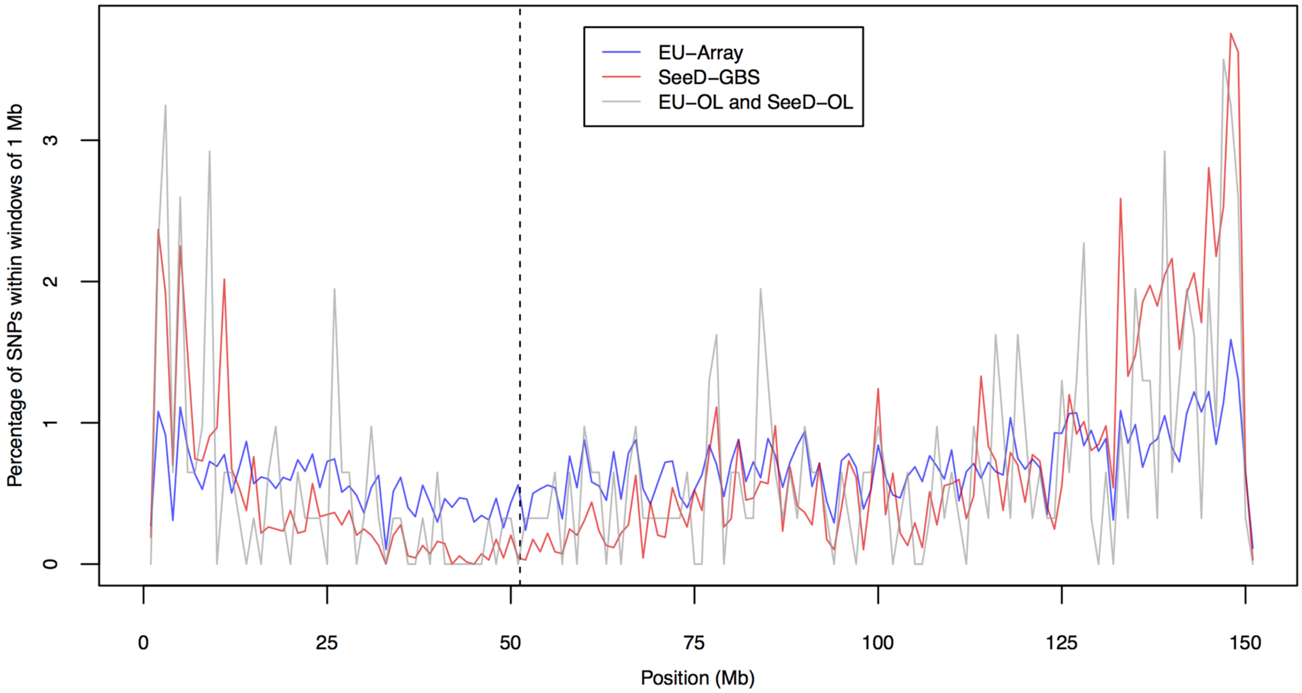

Figure S1 Distribution of SNPs on chromosome 10. The relative percentage of SNPs located in non-overlapping windows of 1 Mb are plotted along chromosome 10. The dashed vertical line indicates the centromere. *blue*: EU-Array, *red*: SeeD-GBS, *gray*: EU-OL and SeeD-OL.

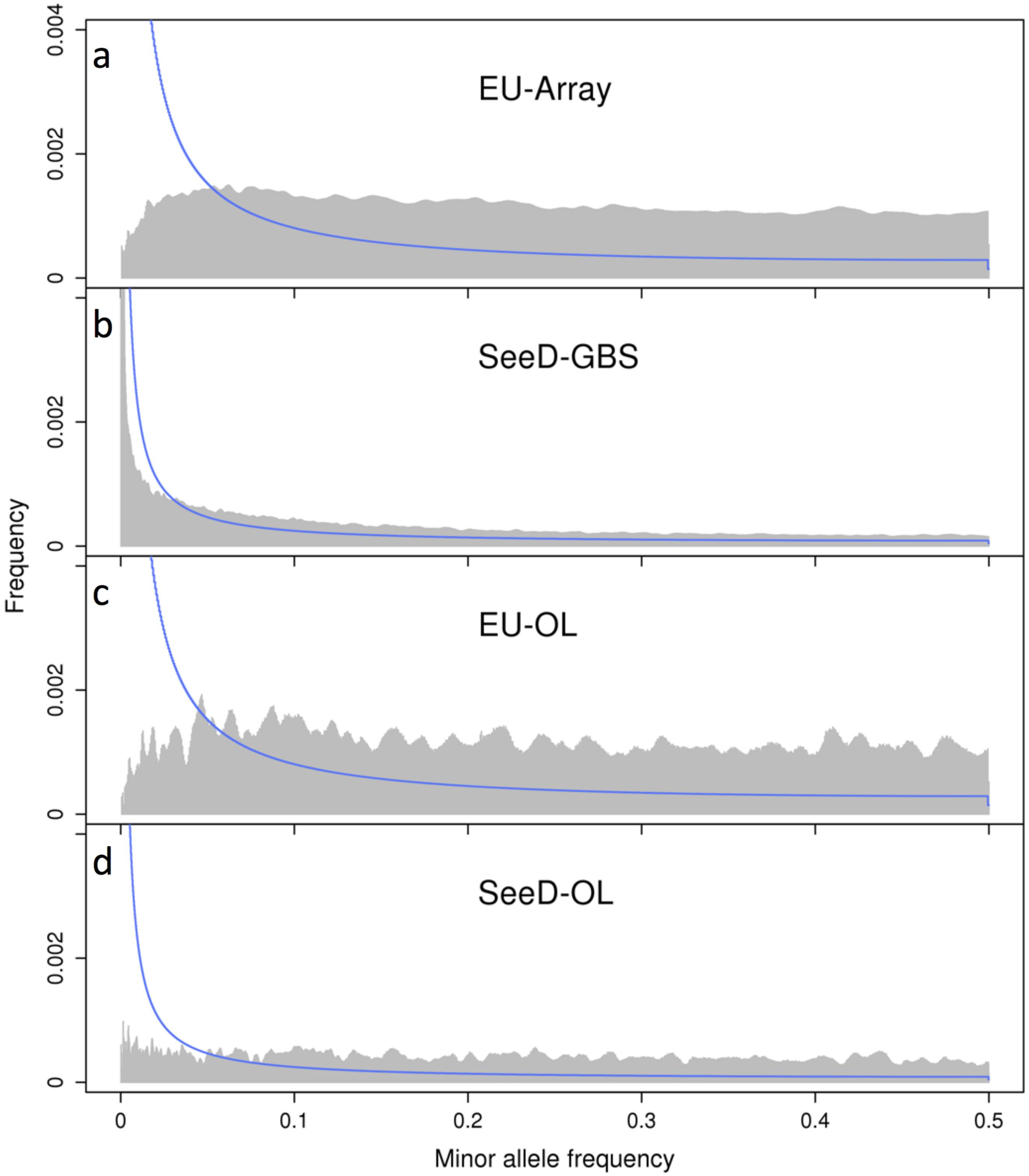

Figure S2 Folded site frequency spectra of the investigated datasets. Histograms of the frequencies of SNPs (y-axis) with a particular minor allele frequency (x-axis) were calculated by randomly sampling 1,000 times 1,714 and 4,962 non-missing alleles per SNP for EU and SeeD datasets, respectively. The number of sampled alleles per SNP corresponds to the product of the minimum call rate and the number of gametes per dataset. *Blue* curves indicate the expected allele frequency distribution under the neutral model. *a*: EU-Array, *b*: SeeD-GBS, *c*: EU-OL, *d*: SeeD-OL.

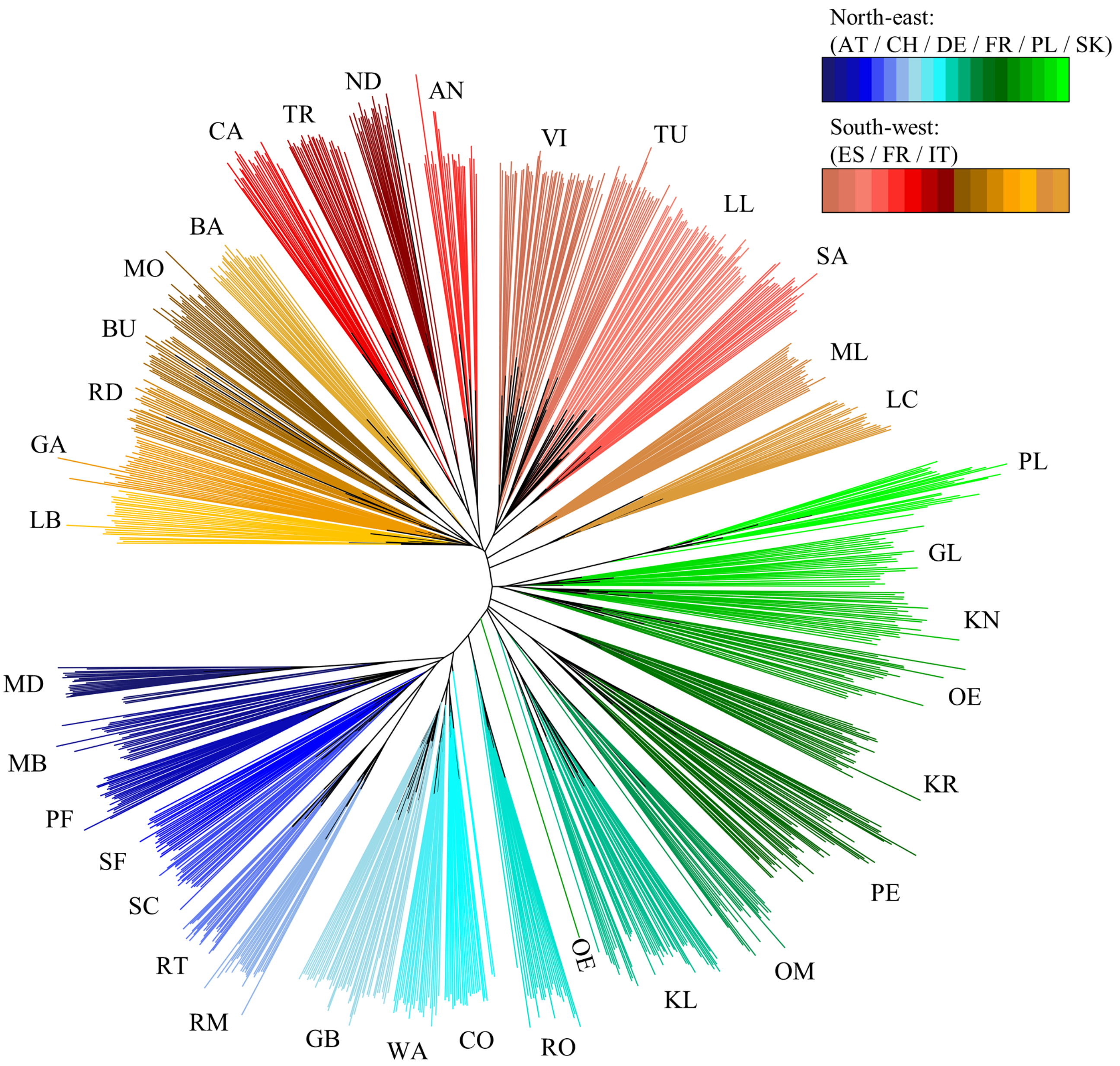

Figure S3 Neighbor joining tree of European landraces based on dataset EU-Array. Landraces are colored according to **Fig. 1a,** with north-eastern and south-western European landraces in *blue*/*green*, and *red*/*orange*, respectively. Internal branches are colored in *black*. Abbreviations of landraces refer to **Table S1**.

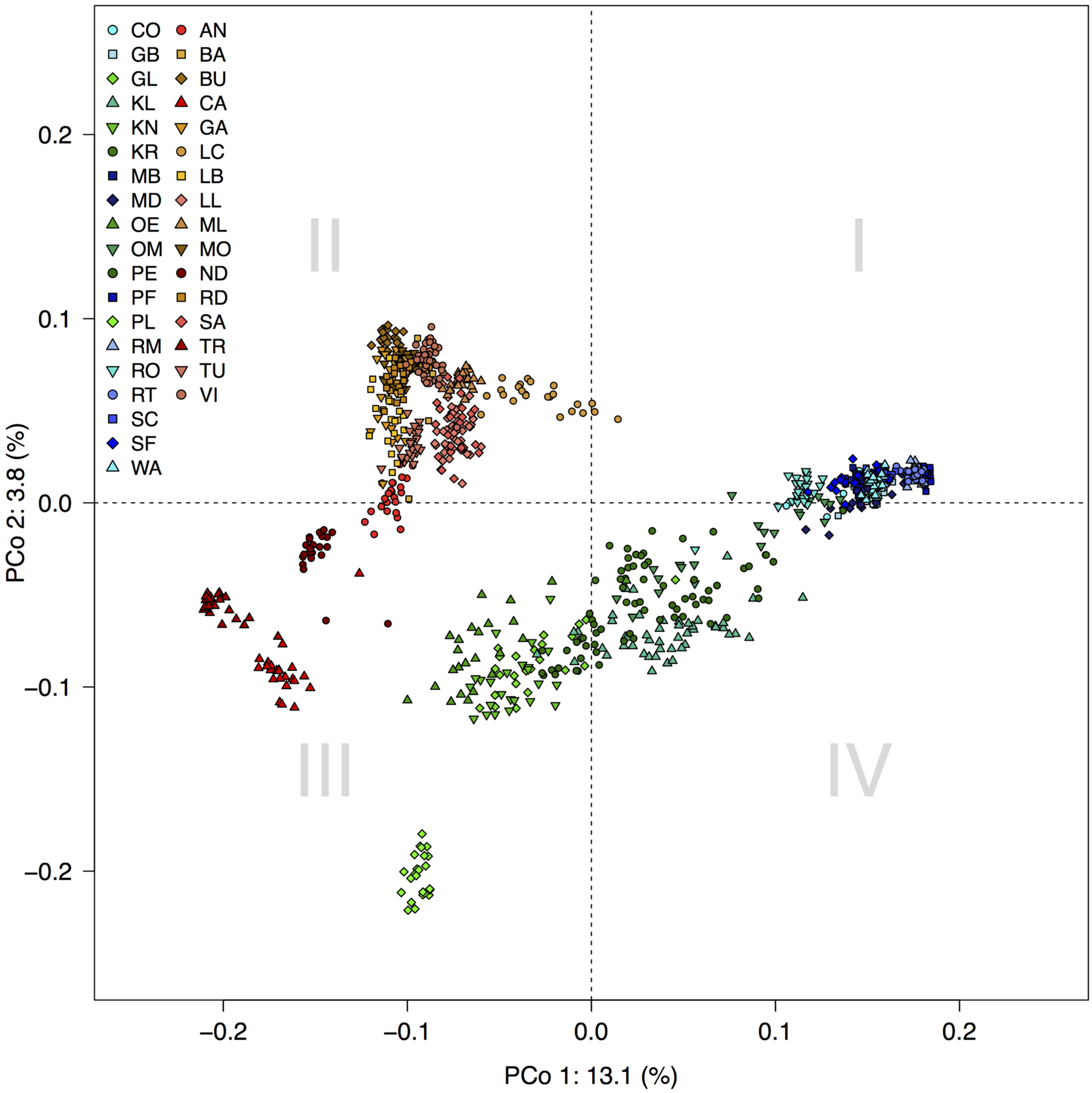

Figure S4 Principal coordinate analysis (PCoA) of European landraces. The PCoA is based on modified Rogers' distances between individuals of dataset EU-Array. Landraces are colored according to **Fig. 1a**. Axis labels show the percentage of explained variance per principal coordinate (PCo). Different symbols are assigned to facilitate the visual differentiation of landraces. Abbreviations of landraces refer to **Table S1**.

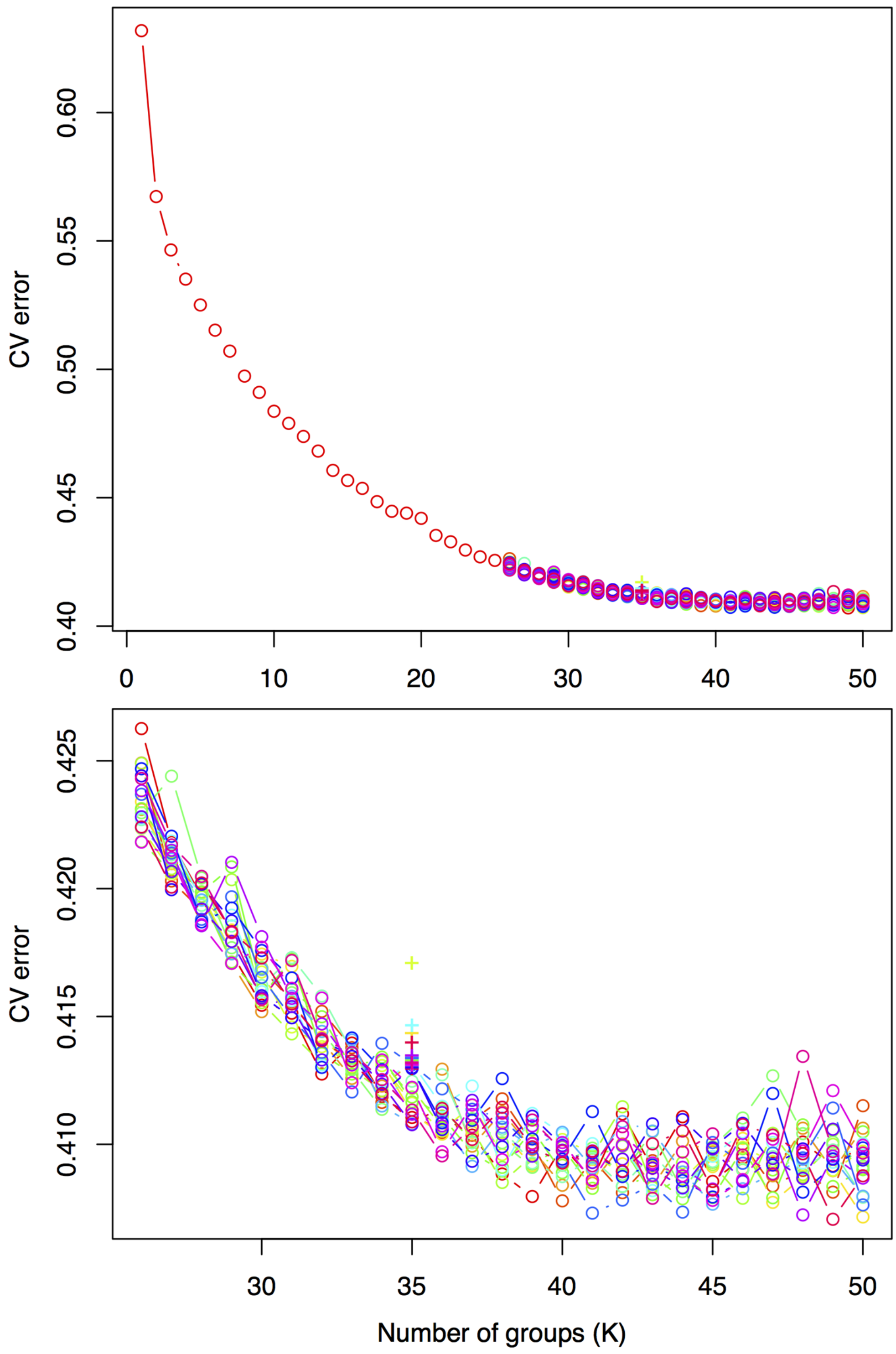

Figure S5 Cross-validation (CV) error plot of the population structure analysis based on EU-Array using ADMIXTURE. Five-fold CV was performed to estimate the most likely number of genetic groups *K*. For each *K* varying from 1 to 25, one run was performed, while for each *K* varying from 26 to 50, 20 runs with different seed settings were conducted as indicated by different colors. CV errors of the supervised analysis with 35 pre-defined groups are represented by pluses. *Top*: Plot for *K* varying from 1 to 50, *bottom*: Plot for *K* varying from 26 to 50.

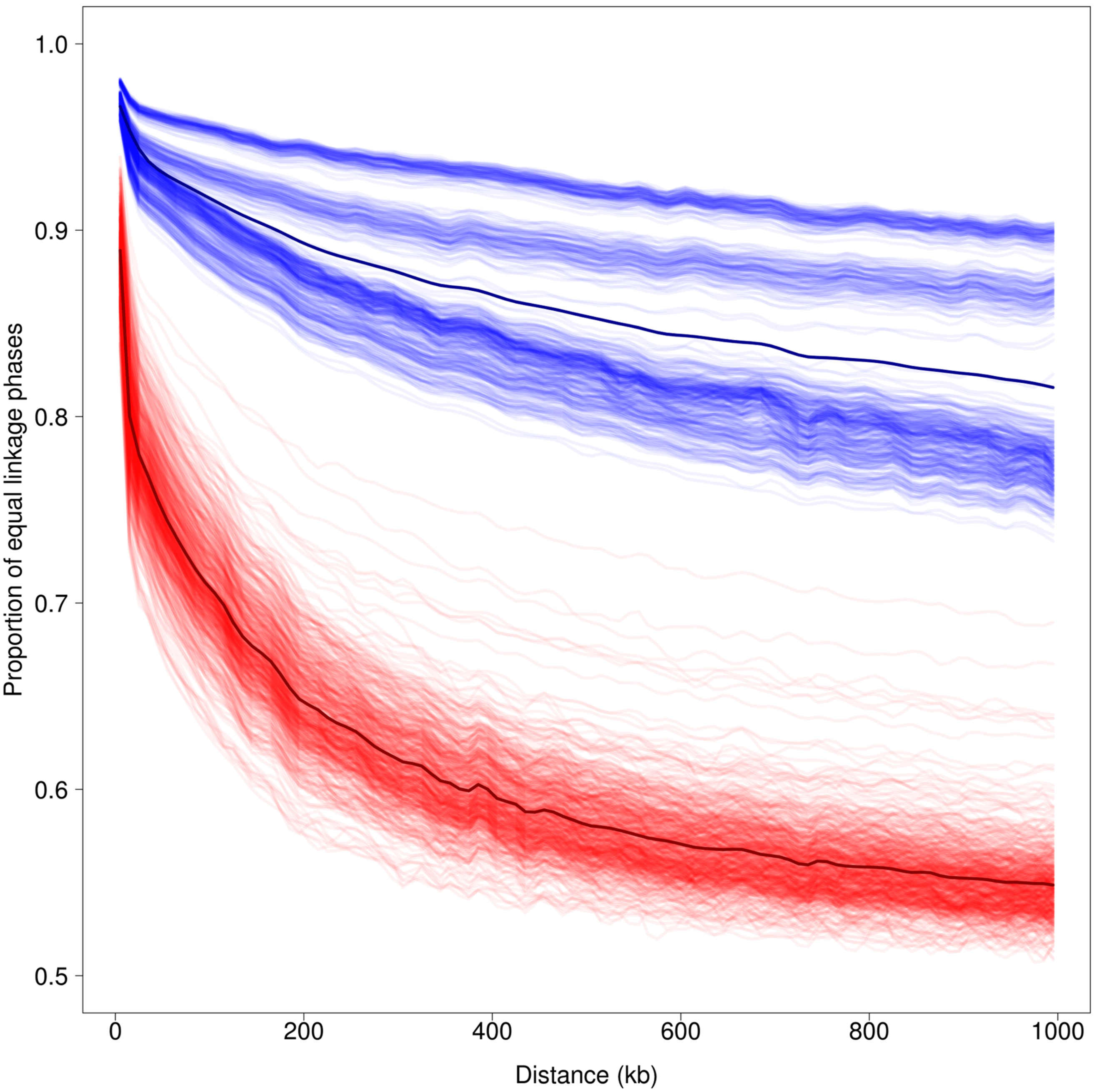

Figure S6 Proportion of marker pairs with equal linkage phase (*PEP*) within and across European maize landraces. Cubic smoothing spline fits are shown for *PEP* between samples within (*blue*) and across (*red*) landraces as a function of physical distance, based on dataset EU-Array. For the within-landrace estimates, 100 times half of the individuals within each of the five landraces with $n_{LR} \geq 46$ (**Table S1**) were randomly sampled and compared to the second half. Across-landrace estimates are based on pairwise comparisons of all 35 landraces. Mean values for within- and across-landrace estimates are shown in *dark blue* and *dark red*, respectively.

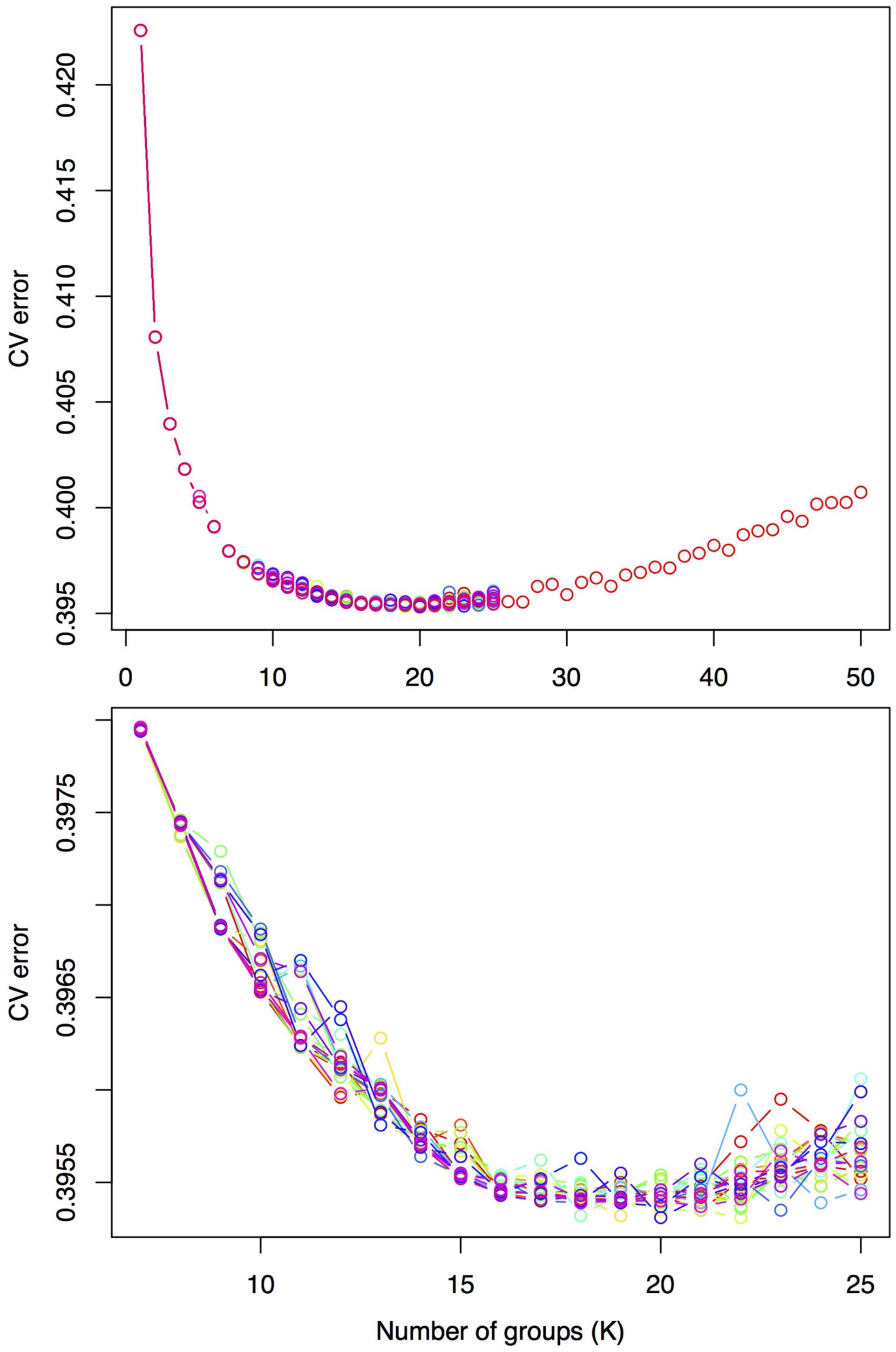

Figure S7 Cross-validation (CV) error plot of population structure analyses based on SeeD-GBS using ADMIXTURE. Five-fold CV was performed to estimate the most likely number of genetic groups *K*. For each *K* varying from 1 to 25, 20 runs with different seed settings were conducted as represented by different colors. For each *K* varying from 26 to 50, one run was performed. *Top*: Plot for *K* varying from 1 to 50, *bottom*: Plot for *K* varying from 7 to 25.

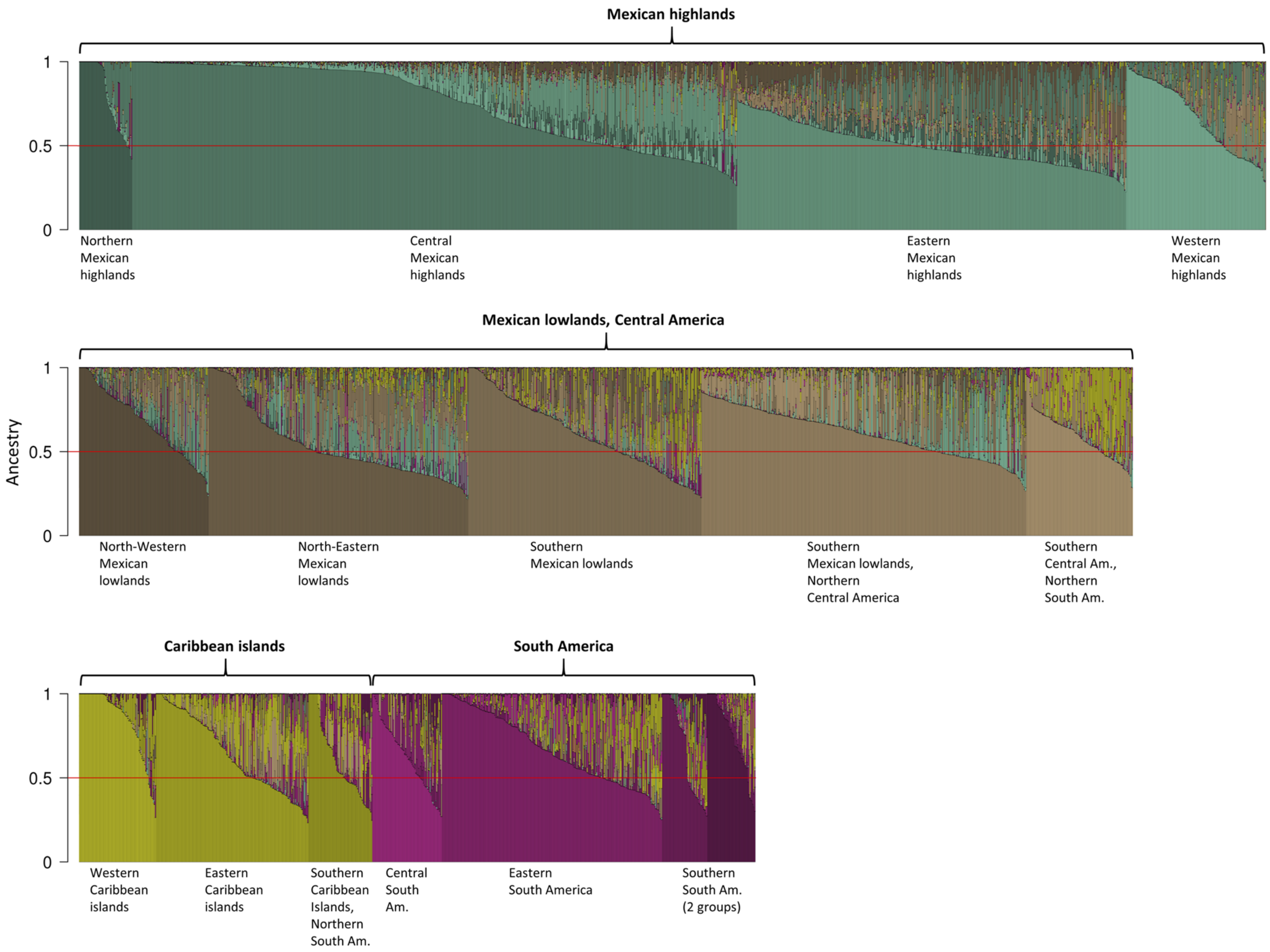

Figure S8 Population structure in American landraces. Genetic groups identified based on dataset SeeD-GBS as revealed by the analysis using ADMIXTURE for the most likely number of 16 genetic groups. Bar plots indicate the relative ancestral composition of individuals, and the red horizontal line an ancestry proportion of 50%. The geographical origin of individuals from the different groups is indicated above and below the structure plot.

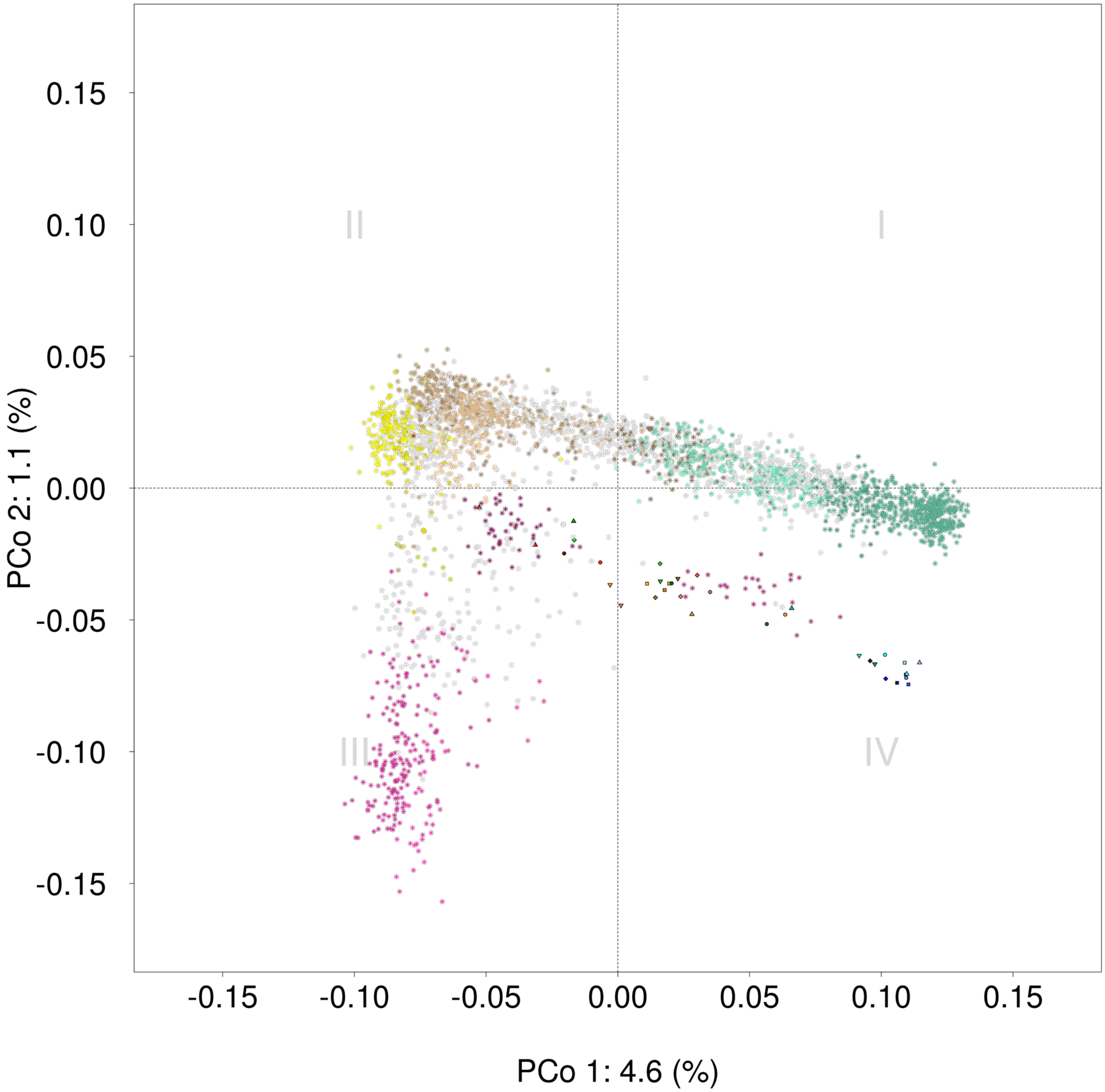

Figure S9 Principal coordinate analysis (PCoA) of European and American landraces. The PCoA is based on modified Rogers' distances between individuals using the combined dataset of SeeD-OL and one representative of each of the 35 landraces sampled from EU-OL. The axis labels show the percentage of explained variance per principal coordinate (PCo). Landraces are colored according to **Fig. 1**. Symbols of European landraces are according to **Fig. S4**, American landraces are represented by stars.

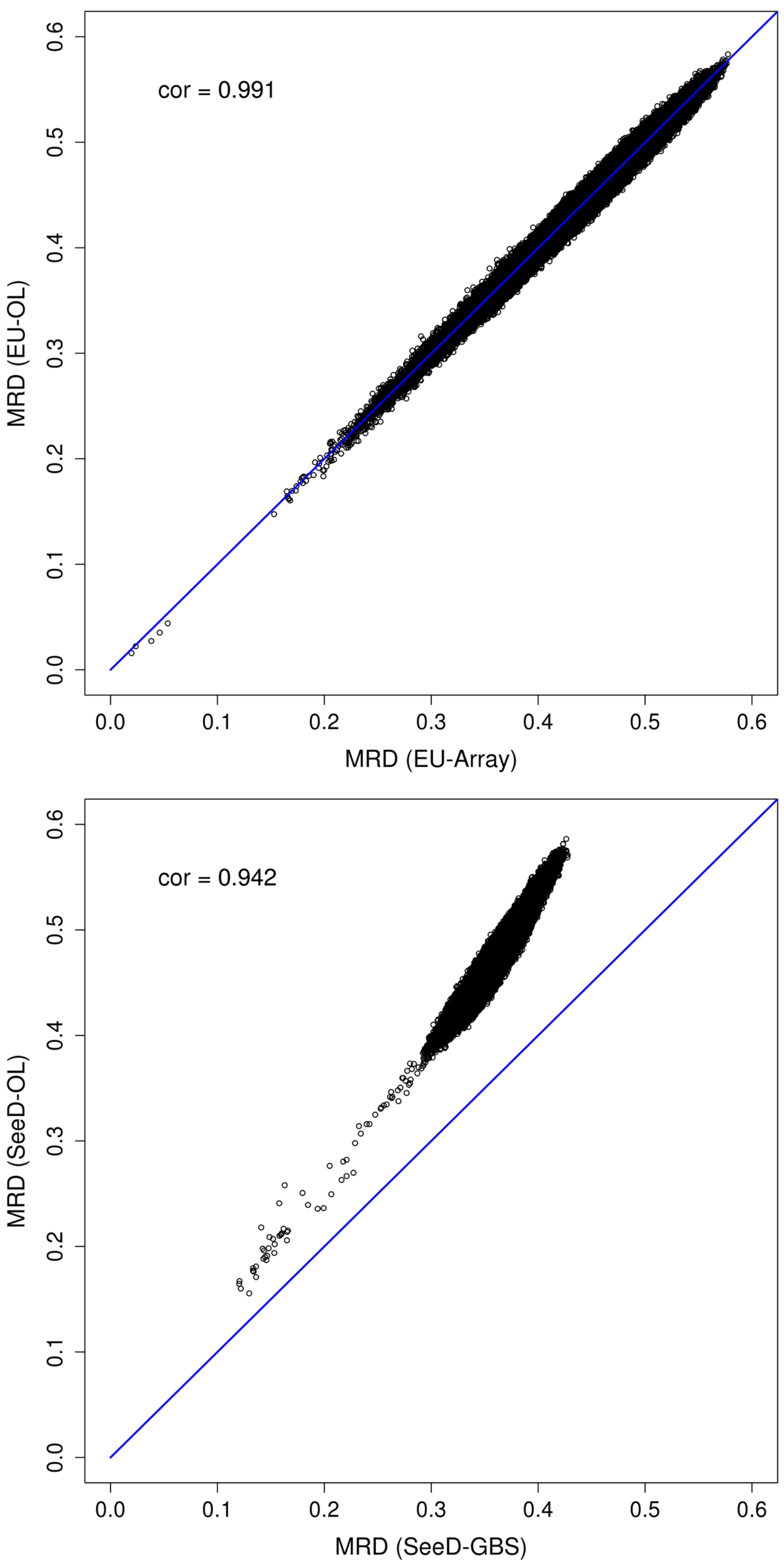

Figure S10 Correlation between modified Rogers' distances obtained by different marker sets. Each data point represents one pair of individuals of the European (EU-Array and EU-OL; *top*) and American (SeeD-GBS and SeeD-OL, *bottom*) datasets.

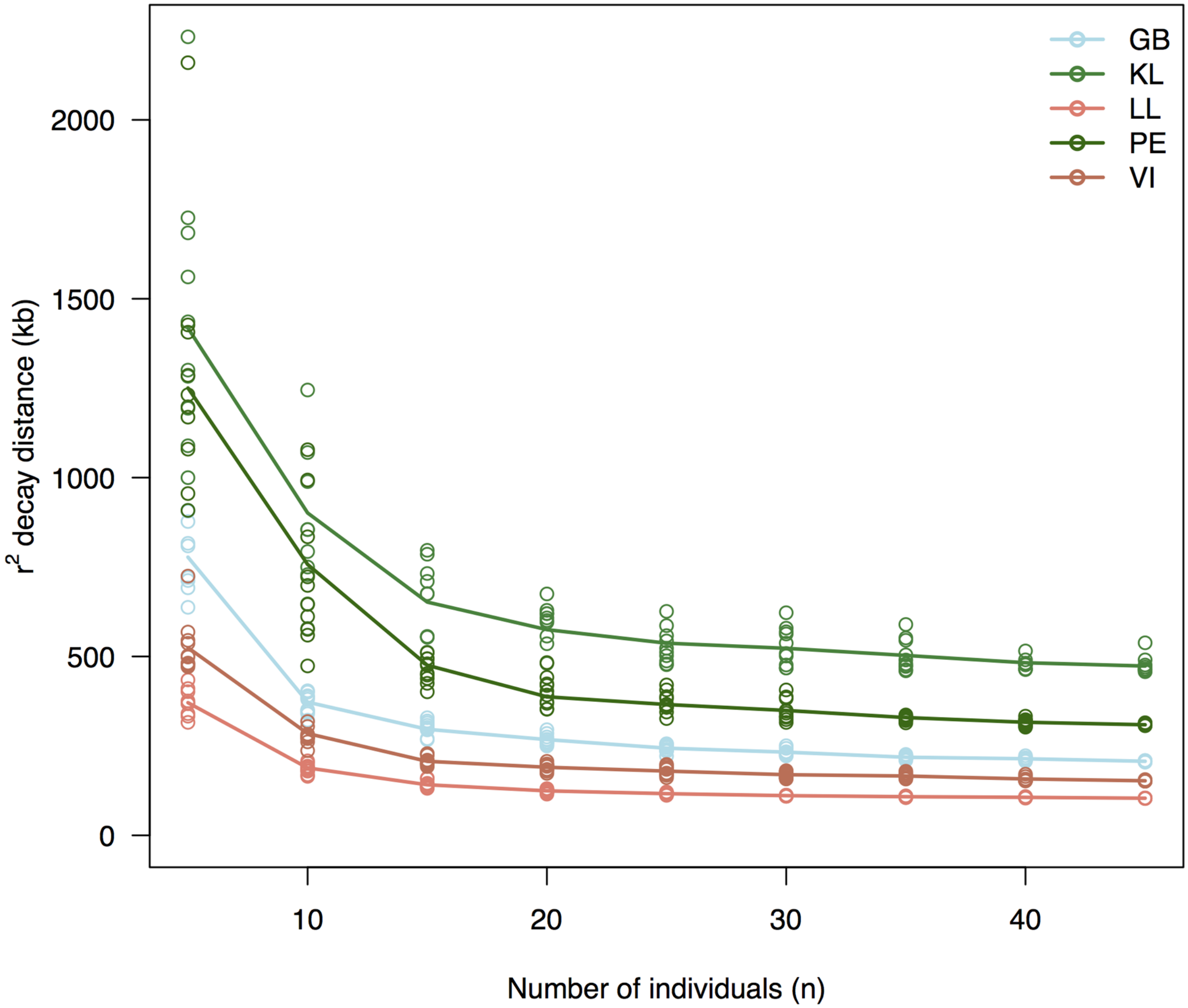

Figure S11 Effect of sample size on LD estimation. For the five landraces of dataset EU-Array with $n_{LR} \geq 46$ (**Table S1**), average LD decay distances in kb for $r^2 = 0.2$ were calculated for samples of 5 to 45 individuals, in steps of 5. Curves show cubic smoothing spline fits for 10 random repeats per landrace.

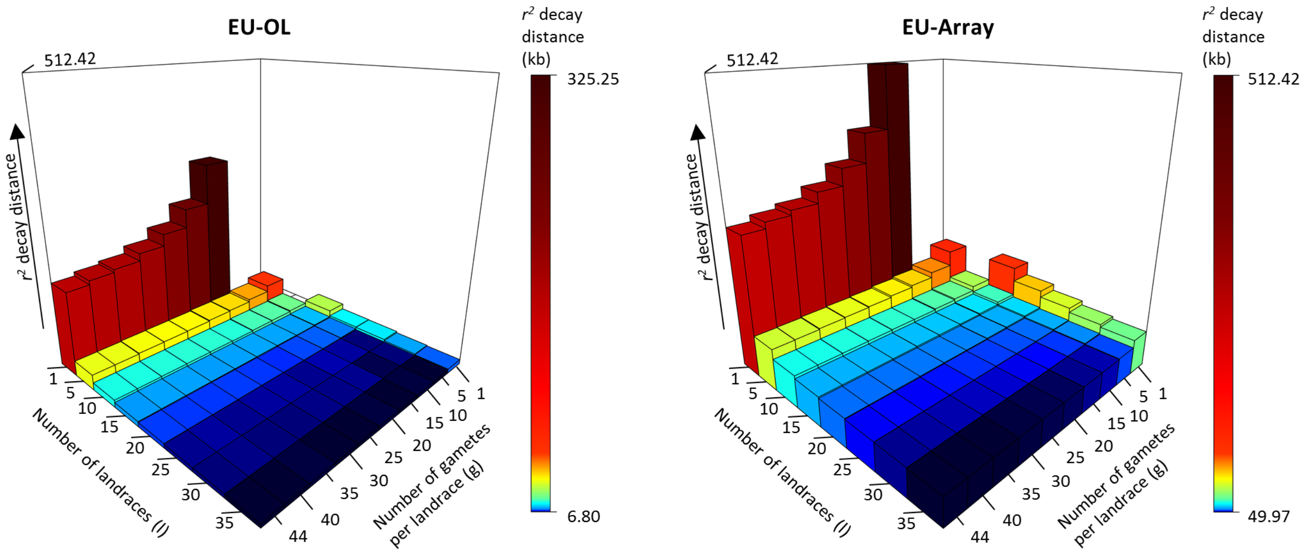

Figure S12 Effects of sample size, sample composition and marker distribution on LD decay distances. Average LD decay distance in kb for r^2 = 0.2 were calculated using non-linear regression for sampling schemes varying in the number of landraces *l* and the number of gametes *g* per landrace. Estimations were performed based on EU-OL (*left*) and EU-Array (*right*). Bars and plot-specific color schemes represent the average r^2 decay distance for 10 random samples per *l* x *g* class.
